# Transparent Glass-Ceramics Produced by Sol-Gel: A Suitable Alternative for Photonic Materials

**DOI:** 10.3390/ma11020212

**Published:** 2018-01-30

**Authors:** Giulio Gorni, Jose J. Velázquez, Jadra Mosa, Rolindes Balda, Joaquin Fernández, Alicia Durán, Yolanda Castro

**Affiliations:** 1 Instituto de Cerámica y Vidrio (CSIC), 28049 Madrid, Spain; ggorni@icv.csic.es (G.G.); josvel@icv.csic.es (J.J.V.); jmosa@icv.csic.es (J.M.); 2 Departamento de Física Aplicada I, Escuela Superior de Ingeniería, Universidad del País Vasco UPV/EHU, 48940 Bilbao, Spain; rolindes.balda@ehu.eus (R.B.); joaquin.fernandez@ehu.es (J.F.); 3 Centro de Física de Materiales UPV/EHU-CSIC, 20018 San Sebastian, Spain

**Keywords:** sol-gel, oxyfluoride glass-ceramics, nanocrystal, optical properties

## Abstract

Transparent glass-ceramics have shown interesting optical properties for several photonic applications. In particular, compositions based on oxide glass matrices with fluoride crystals embedded inside, known as oxyfluoride glass-ceramics, have gained increasing interest in the last few decades. Melt-quenching is still the most used method to prepare these materials but sol-gel has been indicated as a suitable alternative. Many papers have been published since the end of the 1990s, when these materials were prepared by sol-gel for the first time, thus a review of the achievements obtained so far is necessary. In the first part of this paper, a review of transparent sol-gel glass-ceramics is made focusing mainly on oxyfluoride compositions. Many interesting optical results have been obtained but very little innovation of synthesis and processing is found with respect to pioneering papers published 20 years ago. In the second part we describe the improvements in synthesis and processing obtained by the authors during the last five years. The main achievements are the preparation of oxyfluoride glass-ceramics with a much higher fluoride crystal fraction, at least double that reported up to now, and the first synthesis of NaGdF_4_ glass-ceramics. Moreover, a new SiO_2_ precursor was introduced in the synthesis, allowing for a reduction in the treatment temperature and favoring hydroxyl group removal. Interesting optical properties demonstrated the incorporation of dopant ions in the fluoride crystals, thus obtaining crystal-like spectra along with higher efficiencies with respect to xerogels, and hence demonstrating that these materials are a suitable alternative for photonic applications.

## 1. Introduction

Phosphor materials emit light under exposure to external stimulation, such as an electron beam, light at different wavelengths, voltage or electric field, etc. These materials are widely applied in light-emitting diodes, solar cells, sensing, catalysis, integration in photovoltaic devices, and more recently in biosensing, bioimaging, or medical diagnosis [1,2,3,4,5,6]. 

The optical properties of these materials can change drastically depending on the processing parameters. Thus, in the past two decades, interest in studying the synthesis and processing of nanophosphors to develop luminescent materials with high efficiency has increased. The properties of existing devices can be improved by including luminescent phosphors and/or nanocrystalline oxyfluorides doped with lanthanide ions (Ln^3+^). Ln^3+^ ions are commonly used as active ions because they show emissions in a wide spectral range, from UV to NIR, and their use in telecom, lasers, lightening, etc. makes them indispensable nowadays. Moreover, their most stable oxidation state is 3+, consisting of partially filled inner 4f levels screened by outer 5s and 5p orbitals. This allows for maintaining emission energies centered near the same values even in different hosts, making them suitable for applications where certain wavelengths are required. Several studies and applications deal with nanoparticles doped with Ln^3+^ ions suspended in liquid phase or with phosphor powders. However, for many applications as laser materials, waveguides and optical fibers, lightening devices, etc., solid and easy-to-handle samples are required. 

In particular, oxyfluoride nano-glass-ceramics (OxGCs) are attractive materials for photonic applications, because they combine the very low phonon energy of fluoride nanocrystals (NCs) (300–450 cm^−1^) with the high chemical and mechanical stability of oxide glass matrices, especially alumino-silicate ones [7,8,9]. Moreover, such materials can be cast in several forms to obtain the desired device. However, to maintain the transparency and avoid Rayleigh scattering produced by the quite big difference of refractive index between the oxide glass matrix and fluoride crystals, precise control of the crystal size is mandatory. Hence, to obtain materials with high transparency in the UV-Vis range, NCs with a size lower than 40–50 nm are required. 

The pioneering work about OxGCs, published in 1993 by Wang and Ohwaki [10], in which Pb_x_Cd_1−x_F_2_ fluoride NCs doped with Er^3+^ and Yb^3+^ ions were precipitated in a silica-aluminate glass matrix after a proper heat treatment, showed an increase in the Up-Conversion (UC) emission of GCs with respect to precursor glass. Since that paper, the number of publications about transparent GCs has grown exponentially. In the last few decades, many compositions have been studied with the aim of obtaining different crystal phases and using different Ln^3+^ ions as dopants to obtain enhanced linear and nonlinear optical processes. 

The usual method to prepare OxGCs is traditional glass melt-quenching (MQ). This method allows for obtaining several compositions; however, the most studied ones are those based on alumino-silicate matrices, even though there are also some studies of phosphate and fluoride GCs [11,12]. According to the definition of GCs [13], “*Glass-ceramics are ceramic materials formed through the controlled nucleation and crystallization of glass*”. In general, for MQ materials, the crystallization process is performed at temperatures slightly higher than T_g_ (T_g_ + 20–100 °C) to ensure the growth of crystals with a nanometric size that permits maintaining transparency. Moreover, phase separation commonly acts as a precursor for crystallization and the typical crystallization mechanism is a diffusion-controlled process.

The MQ method presents some drawbacks, most of them related to the high temperatures involved during the glass melting. In fact, high melting temperatures (1400–1700 °C) cause relevant fluorine loss, up to 30–40%, thus limiting the final content of crystal phase that can be obtained and resulting in uncontrollable compositions with respect to fluorine and Ln^3+^ ions. In addition, due to quite common phase separation of the precursor glass due to fluorine immiscibility in oxide glass matrices, it is a challenge to prepare high-quality samples for applications as laser devices or any other that requires high optical quality. Furthermore, heat treatments, in general, are quite long (from 3 h up to 120 h) and thin films cannot be directly obtained, thus limiting the possibility of application as integrated devices. Despite these disadvantages, the MQ method demonstrated the possibility of obtaining OxGCs as bulk materials with enhanced optical properties with respect to precursor glass, and in recent years, the preparation of novel OxGC optical fibers has also been achieved [14,15,16,17]. Therefore, MQ is still the most used processing method to obtain OxGCs and is also scalable to an industrial level. Indeed, transparent GC containing up to 75% of crystal fraction, with a crystal size below 40 nm, are extensively used in telescope mirrors [18].

To overcome previous limitations without giving up the field of OxGCs, many researchers tried different processing methods. In particular, the Sol-Gel (SG) route has been recognized and indicated as a promising alternative process to obtain transparent OxGCs. In fact, SG is a handy, flexible, and quite cheap method to fabricate novel and innovative materials at temperatures much lower than those used for MQ materials. Such low temperatures allow for introducing a higher amount of fluoride NCs with much better dispersion than in MQ compositions. Moreover, because SG is a chemical process using a bottom-up approach, high homogeneity can be obtained with no phase separation detected in SG glasses or GCs.

Another important feature of SG is the versatility of the processing method that permits obtaining thin films, powders, and bulk materials. However, many fewer papers about SG OxGCs are reported in the literature compared to those describing materials prepared by MQ [8]. This is mostly associated with the requirement of the optimization of the synthesis method, and in many cases the starting precursor can also affect the final crystal phase. Most of the materials studied have a simple composition that can be easily summarized with the formula:

(100 − x)SiO_2_-x M_1_F_2_/M_1_F_3_/M_1_M_2_F_4_/M_1_M_2_F_5_,

where M_1_ and M_2_ are alkaline, alkaline-earth metals, or lanthanide elements, respectively. More complex glass matrices, where SiO_2_ is partly substituted with Al_2_O_3_, are also reported in the literature but are quite rare, and even more complex structures have also been prepared but for different applications as biomaterials [19]. 

SG synthesis typically involves the hydrolysis and polycondensation of metal salts or metal-organic precursors, such as tetraethyl orthosilicate (TEOS), in an alcoholic medium. The active phase and Ln^3+^ ions are prepared separately using acetates, nitrates, or chlorates as precursors and dissolved using a fluorine acid. The mixing of both solutions, followed by a controlled crystallization, allows for obtaining transparent OxGCs.

Even though the SG method offers potential advantages, they have not been fully exploited, and all the papers published to date describe the preparation of similar compositions containing nominal small crystal fractions (5–10 mol %), with the real crystal content not being estimated. Furthermore, in many cases only slight changes are added to the already published compositions, the main modifications consisting of changing the dopants and/or their concentration. 

In the following sections the most relevant results for several Ln^3+^ doped OxGCs for optical applications studied to date and prepared by SG are summarized. The results are separated into two sections depending on the crystal phase. A third section regarding oxides and oxychlorides GCs has also been added, while in the last sections we will present the results and improvements introduced by our group in the last five years.

### 1.1. Alkaline-Earth Oxyfluoride Glass-Ceramics, M_1_F_2_ (M_1_ = Mg, Pb, Sr, Ca, Ba), M_1_M_2_F_4_ (BaMgF_4_) 

Alkaline-earth fluoride GCs, with the formula M_1_F_2_ (M = Mg, Pb, Sr, Ca, Ba), are useful for photonic applications due to their high optical transparency in a wide spectral range, from (UV) ultraviolet to infrared (IR). MgF_2_-based materials have been extensively studied for different applications, for example as protective coatings on glass optics due to their low chemical reactivity, low refractive index (1.38), and scratch- and weather-resistance. Even though this review is centered on materials with luminescence properties, MgF_2_ materials deserve special attention because they have been among the first fluorides produced by SG. Even more interesting is that the synthesis of MgF_2_ introduced important novelties in the synthesis of OxGCs.

**Figure 1:**
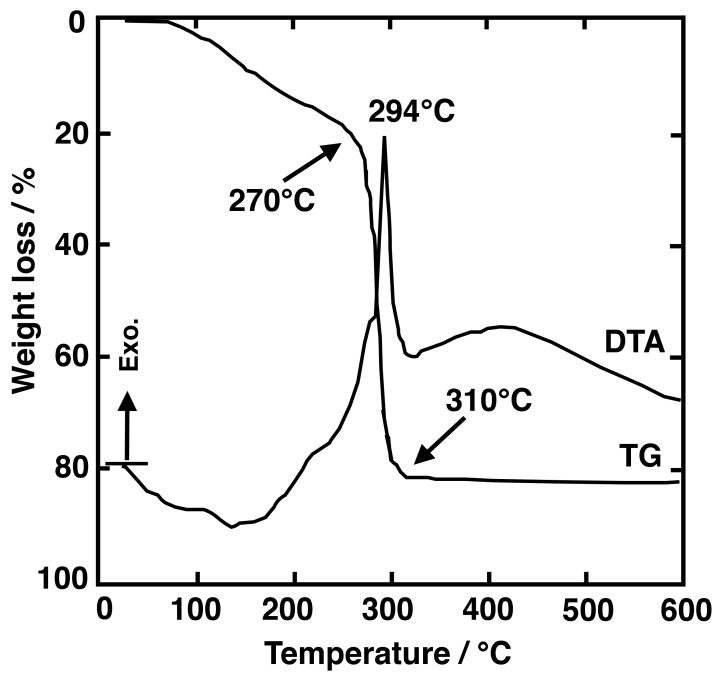
TGA/DTA curve of the MgF_2_ gel obtained by drying the sol at 80 °C. Figure modified from Figure 4 of [22].

One of the first works reported about MgF_2_ NCs and SiO_2_-MgF_2_ materials prepared by SG as bulk and thin films dates back to 1996 [20]. Mg(OCH_3_)_2_ and HF were used as Mg and F sources, respectively, to obtain MgF_2_ sol. SiO_2_-MgF_2_ sols were also prepared with ratios of (mol %) 20:80 and 40:60, using tetramethyl orthosilicate (TMOS) as the SiO_2_ precursor. X-ray diffraction (XRD) showed that MgF_2_ crystals precipitated in both sol and glass samples, but the crystal growth in SiO_2_-MgF_2_ samples was dramatically slowed (~15 nm) in comparison with that of pure MgF_2_ samples (~115 nm). For 20SiO_2_:80MgF_2_ samples, the crystal size maintained the same value. MgF_2_ NCs have been obtained after treatment at different temperatures following the procedure described in [21]. In 1997 Fujihara et al. [22] reported on MgF_2_ thin films using trifluoracetic acid (TFA) and Mg(OC_2_H_5_)_2_ as a fluorine or magnesium precursor, respectively, with a molar ratio 1:2.6. The most important innovation is the use of TFA as a fluorine precursor, together with an explanation of the fluoride NCs’ formation. It was assumed that Mg(OC_2_H_5_)_2_ complexes were formed in the xerogel and upon heat treatment, Mg^2+^ ions react with thermally activated fluorine in CF_3_COO^−^ ions to form MgF_2_ crystals. This decomposition reaction was accompanied by a huge weight loss, around 75%, as confirmed by Differential Thermal Analysis (DTA) (Figure 1). Some years later it was demonstrated that, thanks to the screening effect of CF_3_COO ions of TFA, metal cations with oxidation state 2+ or 3+ are prevented from being incorporated in the SiO_2_ matrix, making possible subsequent fluoride NCs’ precipitation upon heat treatment. Therefore, this work can be considered pioneering and a boost towards the preparation of OxGCs by SG.

Another phase extensively studied is PbF_2_. One of the first works associated with SiO_2_-PbF_2_ samples was reported in 2004 by Luo et al. [23]. Bulk materials of composition 90SiO_2_-10PbF_2_ doped with Er^3+^ were prepared using TEOS and TFA as the SiO_2_ or fluorine precursor, respectively. The molar ratio of precursors TEOS:CH_3_CH_2_OH:DMF:Pb(CH_3_COO)_2_:TFA:H_2_O:HNO_3_ was 1:2:2:0.1:0.6:4:0.4. Upon heat treatment at 300 °C and 480 °C β-PbF_2_ NCs precipitated in the glass matrix. Er^3+^ ions seemed to be segregated at the surface of the crystallites and hindered the growth of PbF_2_ NCs, thus delaying crystallization and reducing the crystal size from 20 to 9 nm. Other authors reported on UC measurements in bulk materials of the same composition doped with 0.1Er^3+^-0.3Yb^3+^ [24]. A TEOS:EtOH:H_2_O:CH_3_COOH ratio of 1:4:10:0.5 was used for the SiO_2_ sol, while the ratio Ln(CH_3_COO)_3_:TFA was 1:4. UC bands showed well-resolved Stark components indicating the incorporation of Ln^3+^ ions into PbF_2_ crystals. Results for the same composition doped with Tm^3+^-Yb^3+^-Er^3+^ and Tm^3+^-Yb^3+^-Ho^3+^ were also reported [25]. In both cases, the color tunability of UC luminescence was obtained, allowing white UC generation. Following the same synthesis and composition of Luo et al. described before, Szpikowska-Sroka et al. published some papers about OxGCs with different dopants [26,27,28]. After heat treatment, Eu^3+^ and Tb^3+^ ions showed an increase in the emission spectra in GC samples with respect to the xerogels due to the incorporation of these ions into the NCs. In fact, the asymmetry ratio, *R*, described as the ratio between the two visible emissions I(^5^D_0_-^7^F_2_)/I(^5^D_0_-^7^F_1_) of Eu^3+^, gives information about the nature of the environment surrounding the ion. Moreover, the effect of the TFA on the optical properties was also analyzed, showing the best result for a Pb/TFA ratio of 5 and corresponding to a crystal fraction of 3 wt %. 

90SiO_2_-10SrF_2_ transparent GCs doped with ErF_3_ (1 mol %) were prepared by Yu et al. in 2006 [29]. Samples treated at 300 °C already showed the precipitation of SrF_2_ crystals with a size of 10 nm. The same crystal size is maintained for heat treatment up to 800 °C. However, upon excitation at 378 nm no Er^3+^ emission was detected unless for samples treated at 800 °C. The authors attributed this phenomenon to the presence of –OH groups that quench the luminescence. Two years later, the same authors prepared transparent bulk samples of composition 90SiO_2_-10SrF_2_-0.5ErF_3_ and 97.5(90SiO_2_-10SrF_2_)-2.5Al_2_O_3_-0.5ErF_3_ (mol %) [30]. Al_2_O_3_ was added as the nitrate, with Al^3+^ acting as a glass network former, replacing Si^4+^. The introduction of Al^3+^ to the SiO_2_ network causes a decrease in non-bridging oxygens, hindering the crystallization of fluoride NCs. The SiO_2_-Al_2_O_3_ glass matrix showed higher transparency in the UV region due to lower pore content and the final optical properties were better for this composition treated at 1000 °C. Very intense and visible UC luminescence was observed for GC based on SiO_2_-Al_2_O_3_ matrix, as compared to the one with only SiO_2_.

The first SiO_2_-CaF_2_ transparent GC prepared by SG was reported in 2007 by Zhou et al. [31]. 95SiO_2_-5CaF_2_ bulk samples doped with 1 mol % ErF_3_ were treated at 900 °C, allowing the precipitation of CaF_2_ crystals of 20 nm in size, homogeneously distributed in the amorphous SiO_2_ matrix. Er^3+^ incorporation in the crystal phase was tested by Energy-Dispersive X-ray Spectroscopy (EDXS). Only Red UC emission was detected upon 980 nm excitation. Georgescu et al. prepared 89SiO_2_-5CaF_2_-5YbF_3_-1ErF_3_ (mol %) GCs upon heat treatment at 800 °C [32]. A solid solution of (Ca_1−x_Ln_x_)F_2+x_ crystals was observed by XRD, and both red–green and Violet-UV UC were observed when exciting the samples at 973 nm. The process was described as a two-photon and three-photon absorption process for visible and UV emissions, respectively, upon IR excitation. On the other hand, a different approach was used to prepare 95SiO_2_-5CaF_2_ GCs [33]. Er^3+^-doped CaF_2_ crystals were previously synthesized by co-precipitation using Er(NO_3_)_3_, Ca(NO_3_)_2_, and ammonium bi-fluoride NH_4_HF_2_ as precursors. Then, NCs were mixed to SiO_2_ sol, dried at 50 °C during one month and heat-treated to obtain bulk materials. The authors considered that conventional SG route does not allow control of the size and quantity of crystals as well as the Ln^3+^ concentration into the NCs. The optical results showed that the UC emissions increased when passing from xerogels to GCs, the corresponding lifetimes of the ^4^F_9/2_-^4^I_15/2_ emission being 1.3 μs and 1.7 ms, respectively. This effect was ascribed to the incorporation of Er^3+^ into CaF_2_ NCs with phonon energy around 460 cm^−1^.

**Figure 2:**
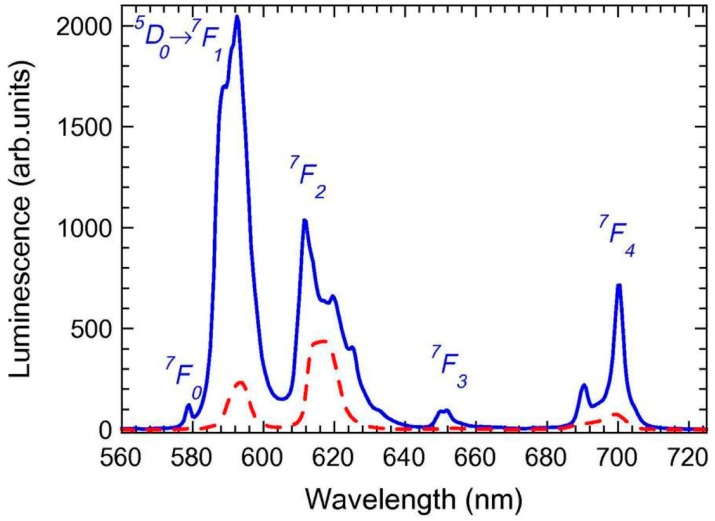
PL spectra recorded on Eu^3+^-doped xerogel (dotted curve) and GC (solid curve) using λex. = 394 nm [35].

Another interesting host for Ln^3+^ ions is BaF_2_ due to its very low phonon energy, around 320 cm^−1^. The first SiO_2_-BaF_2_ SG material was reported in 2006 by Chen et al. [34]. 95SiO_2_-5BaF_2_ bulk materials were prepared and doped with 0.5 and 1 Er^3+^ (mol %). BaF_2_ crystals, 2–4 nm in size, precipitated in the glass matrix after heat treatment at 300 °C. Up to 700 °C, no relevant effects in the crystallization were observed. Over this temperature, the crystal grew up to 10 nm. However, treatment at higher temperature also caused the crystallization of the glass matrix in the form of cristobalite. The incorporation of Er^3+^ ions into the NCs was revealed due to the shift of the XRD peaks toward higher angles in Er^3+^-doped samples and by EDXS and Judd–Ofelt calculations. Photoluminescence (PL) emission was detected only for samples treated at 800 °C and green and red UC emissions were observed upon 980 nm excitation. The same composition and synthesis were used by Secu et al. when doping materials with Pr^3+^, Sm^3+^, Eu^3+^, Dy^3+^, and Ho^3+^ [35,36]. Small BaF_2_ crystals (∼7 nm) were precipitated upon heat treatment at 800 °C for 1 h. No Ln^3+^ emission was observed for xerogels except for Eu^3+^, while for all the GCs typical 4f-4f transitions were observed showing the Stark splitting of the bands (Figure 2). Moreover, the typical Eu^3+^ emissions in GCs indicated that these ions are effectively incorporated into NCs. The Eu^3+^ lifetime, measured at 620 nm, increased from 0.3 to 4.7 ms passing from xerogel to GC.

To conclude this first section, we mention the preparation of 90SiO_2_-10BaMgF_4_ films doped with Eu^3+^ prepared by Fujihara et al. [37]. Upon heat treatment at 750 °C for 10 min small BaMgF_4_ NCs 3 nm in size, were observed by High-Resolution Transmission Electron Microscopy (HRTEM). By N,N-dimethylformamide (DMF) addition a porous film was obtained, while, without DMF the film was dense. DMF addition also favors crystallization, reducing stresses in films [38]. The corresponding PL measurements only showed Eu^2+^ emission, meaning that Eu^2+^ ions are effectively incorporated in the crystal phase, substituting Ba^2+^ ions. Furthermore, Eu^2+^ emission was much stronger for porous films due to higher photon confinement, producing an increase in the absorption process. 

### 1.2. Lanthanides Oxyfluoride Glass-Ceramics, M_1_F_3_ (M_1_ = La, Y, Gd) and M_1_M_2_F_4_, M_1_M_2_F_5_ (M_1_ = Na, K, Li; M_2_ = Gd, Y)

#### 1.2.1. M_1_F_3_

Oxyfluoride GCs in which one element of the NCs former is a Ln^3+^ ion are the most studied compositions. In fact, Ln^3+^ ions are easily replaced by other ions used as dopants, due to their similar ionic radius and equal charge. Moreover, some ions as Gd^3+^ are known to act as sensitizers for other ions as Eu^3+^, Tb^3+,^ and Dy^3+^ [39,40,41], making possible energy transfer (ET) processes and thus making this NC phase attractive for several photonic applications. 

Among all the lanthanide crystals, LaF_3_ is the most studied. Several papers about LaF_3_ OxGCs have been published since 1998, when Fuhijara et al. described the preparation of LaF_3_ thin films on silica substrates [42]. In that work, the authors explained the importance of controlling the synthesis and heat treatment process to obtain high-purity LaF_3_ NCs, avoiding the precipitation of other phases as LaOF. In the following years, the same authors worked extensively in the preparation of SiO_2_–LaF_3_ bulk samples, observing the crystallization of LaF_3_ NCs with a size ranging from 10 to 30 nm [43]. They also studied the influence of TFA on the LaF_3_ NCs’ crystallization, proposing that such formation occurs by the following chemical reaction [44,45,46]:

La(CF_3_COO)_3_→LaF_3_ + (CF_3_CO)_2_O + CO + CO_2_.

However, the authors stated that it is not possible to raise the LaF_3_ content beyond 10 mol % without losing transparency. Therefore, all the papers published after this work concentrated on Fujihara’s synthesis and composition, just changing the active phase. TMOS was partially or totally replaced by TEOS but the same stoichiometric ratios of H_2_O and TFA were used, bringing little innovation to the synthesis and processing of OxGC materials.

The preparation of transparent Eu^3+^ doped SiO_2_-LaF_3_ and SiO_2_-LaOF OxGC films, in which Eu^3+^ ions were incorporated into the LaF_3_ crystals, resulted in interesting PL emissions [47]. Results showed important differences depending on the sintering temperature and, therefore, on the crystals phase. On the other hand, Ribeiro et al. [48] prepared films with the same composition, finding that for heat treatment between 600 and 900 °C both LaF_3_ and LaOF phases appeared. Eu^3+^ ions emissions mainly from the ^5^D_0_ excited state reflected the change in the environment of these ions. They used thin films as planar waveguides at Vis and IR wavelengths, reported losses ~1.8 dB/cm at 632.8 nm. However, the authors considered that the intensity of PL measurements depends on the nature of the crystal phase and the presence of –OH groups that are responsible for PL quenching. At such a high sintering temperature, –OH groups could be eliminated, but fluorine can be lost, producing a silica matrix rich of lanthanide ions and changing the emission spectra. 

LaF_3_ OxGC bulk materials were also prepared by other authors. Biswas et al. [49] described the preparation of transparent 95SiO_2_-5LaF_3_ GC, after densification at 1000 °C, using TEOS instead of TMOS as the SiO_2_ precursor and a TEOS:H_2_O:CH_3_COOH molar ratio of 1:10:0.5. Optical results showed very good UC efficiency from the IR-to-UV region due to the Yb^3+^–Er^3+^ ET process, which is enhanced in GCs, suggesting the incorporation of Er^3+^ ions in the low-phonon environment of LaF_3_ NCs. 

**Figure 3:**
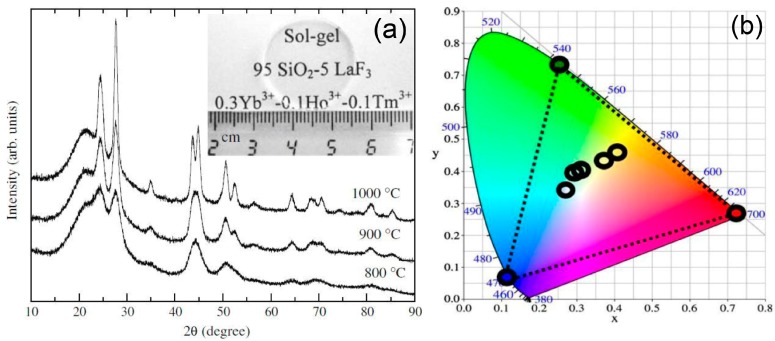
(**a**) XRD patterns of 95SiO_2_-5LaF_3_ GCs at different temperatures and (**b**) CIR standard chromaticity showing the up-conversion emission for GCs co-doped with 0.3Yb^3+^, 0.1Ho^3+^ and 0.1 Tm^3+^ [53].

In order to optimize the optical properties, other authors have described the preparation of bulk GC materials with molar ratios (100 − x)SiO_2_-xLaF_3_ (x = 5 and 10 mol %), doped with (0.1–0.5) mol % of Ln^3+^ ions and heat treated at temperature up to 1000 °C [50,51,52,53,54,55] following a similar synthesis to Biswas et al. Optical results showed that Eu^3+^ ions could be selectively excited and, even more interesting, opened the way for the estimation of the percentage of ions that are incorporated in the NCs, concluding that at least 50% are effectively incorporated in LaF_3_. Another paper [53] described the preparation of transparent 95SiO_2_-5LaF_3_ bulk GC, co-doped and tri-doped with Ln^3+^ ions. Good UC properties after excitation at ~980 nm were observed due to the interionic distance reduction between Ln^3+^ ions, as a consequence of their incorporation into the NCs. Moreover, by increasing the pump power the emitting color was tuned, obtaining white light generation with potential applications in multicolor solid-state displays and optical integrated devices (Figure 3). The incorporation of a high amount of dopant ions, around 75%, in the precipitated LaF_3_ NCs was also reported by Velázquez et al. [54] for Tb^3+^-Dy^3+^ co-doped GCs, showing enhanced UV absorption band and allowing a shift in such emission to the Vis range. 

**Figure 4:**
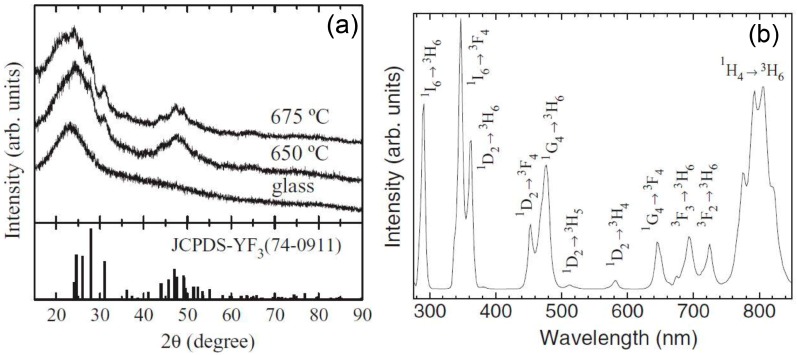
(**a**) XRD patterns of 90SiO_2_-10YF_3_ Ln^3+^-doped GCs heat treated at 650 and 675 °C together with the JCPDS-YF_3_ and (**b**) Up-conversion emission spectrum of GCs co-doped with 1.5 Yb^3+^ and 0.1 Tm^3+^ heat-treated at 675 °C [60].

Other oxyfluoride GCs with special interest for photonics are those containing YF_3_ NCs. This crystal phase can act as a high quantum efficient host lattice for Ln^3+^ ions. Since 1998, when Dejneka published one of the first works on Eu^3+^-doped YF_3_ OxGCs by the MQ method [56], many authors started preparing materials containing this crystal phase. However, it was necessary to wait until 2006 to find one of the first works related to SG bulk materials based on the composition 90SiO_2_-10YF_3_ [57]. The authors precipitated YF_3_ crystallites with sizes around 5 nm from the silica matrix when the xerogel was treated at 400 °C. According to the authors, NCs aggregated to form larger particles. By increasing the temperature to 600 °C, YF_3_ NCs tended to separate without changing their size, finally resulting in a homogeneous distribution in the glass matrix. They proposed that the precipitation of these crystals in the glassy phase induces high stress in the local region, which was reduced when increasing the treatment temperature due to the separation of the NCs from the agglomerates. Méndez-Ramos and co-workers published results based on the same 90SiO_2_-10YF_3_ composition [58,59,60]. In these works, using Ln^3+^ as probe ions of the local environment, the authors found that a large fraction of optically active ions is efficiently embedded into the YF_3_ NCs, 11 nm in size (Figure 4a). When the samples were co-doped with Yb^3+^-Tm^3+^, bright and efficient UC was achieved (Figure 4b), along with intense high-energy emissions in the UV range, due to rare 4- and 5-infrared photon processes. Moreover, co-doping with Ho^3+^ or Er^3+^ ions showed white light generation. 

**Figure 5:**
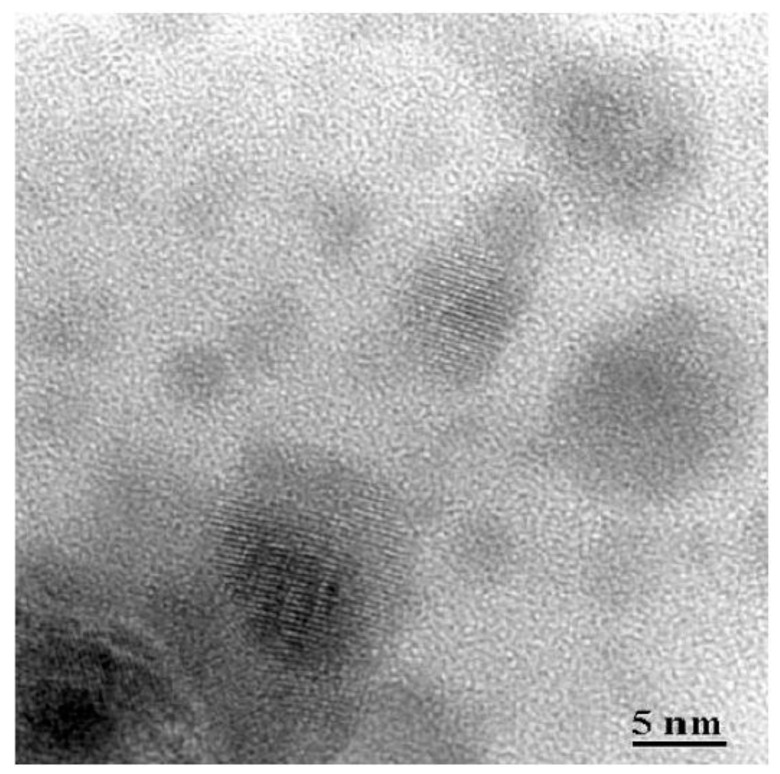
HRTEM image of Eu^3+^ doped SiO_2_-GdF_3_ GC film heat = treated at 400 °C [64].

Differently to LaF_3_ and YF_3_, GdF_3_ is considered a promising host because Gd^3+^ ions can act as s sensitizer for other Ln^3+^ ions and favor some ET processes [61], being suitable for application in Plasma Display Panels (PDPs) and mercury-free fluorescent lamps [62,63]. Only a few papers about SG GdF_3_ OxGCs have been published, with the nominal crystal phase content being less than 10 mol %. Fuhijara et al. reported the synthesis of 90SiO_2_-9GdF_3_ thin films doped with 1 mol % of EuF_3_ [64]. Results confirmed that GdF_3_ NCs, 5 nm in size, precipitated during heat treatment at 300 and 400 °C, Figure 5, but GdOF NCs appeared when the temperature was increased to 500 °C. More recently, Szpikowska-Sroka et al. studied the optical properties of Eu^3+^-doped SiO_2_-GdF_3_ GCs, with a very low composition of active media, around 3–6 mol %, based on Fuhijara’s synthesis [65,66,67]. The PL associated with Eu^3+^ ions in GdF_3_ phase was more efficient due to the low phonon energy of the crystal phase. 

#### 1.2.2. M_1_M_2_F_4_/F_5_

Complex fluoride structures became increasingly interesting in the last decade due to their good optical properties and capability of producing intense and efficient nonlinear optical processes as UC emission or other ET processes. In most cases phosphors were synthesized using SG, solvo-thermal, or co-precipitation methods and the final materials were used as powders. Other works concern the preparation of bulk materials and only few are about thin films. 

Phosphors based on NaYF_4_ crystals have been known since the 1970s for their excellent UC properties when doped with Yb^3+^ and Er^3+^. In particular, NaYF_4_ phosphors’ UC emission was found to be 4–5 times higher than that of LaF_3_ crystals [68]. Kramer et al. proposed a route to synthesize only pure hexagonal NaYF_4_ phosphors [69]. Moreover, transparent NaYF_4_ OxGCs bulk materials have been obtained by the MQ method, showing interesting PL properties even more efficient than those of powder phosphors [70,71].

However, the first work on NaYF_4_ OxGCs prepared by SG was published in 2009 by Yanes et al. [72]. 95SiO_2_–5NaYF_4_ bulk materials doped with 0.1Er^3+^ and 0.3Yb^3+^ were obtained using acetates as precursor and the “Fujihara route”. The ratio between red and green UC emission bands varied as a function of temperature of heat treatment and pump power resulting in color-tunable UC phosphors. The same authors also prepared 95SiO_2_–5NaYF_4_ bulk materials doped with 0.1Eu^3+^ (mol %) [73]. NaYF_4_ face-centered cubic NCs precipitated upon heat treatment between 550 and 650 °C, their size increasing from 5 to 10 nm with the treatment temperature. PL measurements demonstrated the incorporation of Eu^3+^ into the NCs, due to changes of emission bands using a selective excitation, together with an increase in the lifetime. The authors also reported bright white light generation achieved in similar materials tri-doped with Yb^3+^-Ho^3+^-Tm^3+^ [74] and Yb^3+^-Er^3+^-Tm^3+^ [75].

**Figure 6:**
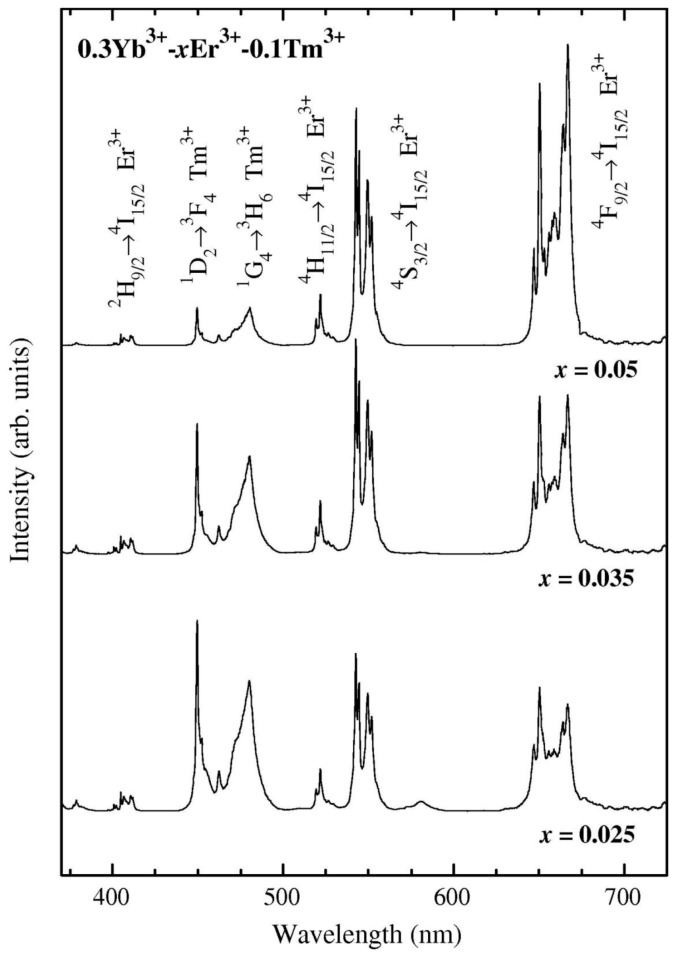
Up-conversion emission spectra of 95SiO_2_-5KYF_4_ GCs co-doped with Yb^3+^-Er^3+^-Tm^3+^ heat-treated at 700 °C [76].

To the best of our knowledge, no KYF_4_ GC or composite materials prepared by MQ have been reported in the literature. The first GC was prepared by Mendez-Ramos et al. using the SG method and co-doping with Yb^3+^-Er^3+^-Tm^3+^ [76]. The processing method was the same used by the authors for other phases such as NaYF_4_, LaF_3_, etc. K, Y, and Ln^3+^ acetates were dissolved using ethanol, TFA, and water, while hydrolyzed TEOS were used as the SiO_2_ precursor, employing the well-known TEOS:EtOH:H_2_O:CH_3_COOH ratio 1:4:10:0.5. KYF_4_ crystals, 14–20 nm in size, were observed treating 95SiO_2_-5KYF_4_ bulk materials between 650 and 700 °C. Very well-resolved PL Stark components were observed for Er^3+^ and Tm^3+^ ions, indicating their incorporation into low-phonon KYF_4_ crystals (Figure 6). UC was obtained upon Yb^3+^ excitation at 980 nm and tunable emission was achieved depending on the dopant concentration and excitation power, allowing white light generation. The same composition was also studied by doping the system with other Ln^3+^ ions [77,78,79]. In all cases, the incorporation of Ln^3+^ ions into cubic KYF_4_ crystals was proved by PL measurements that showed defined Stark components, a better DC and UC process, and an increased lifetime as compared to emissions of Ln^3+^ ions remaining in the glass matrix. The possibility of obtaining both UC and DC simultaneously had interesting applications for a photovoltaic silicon solar cell and white light-emitting diodes. 

Despite some works based on LiYF_4_ OxGCs prepared by the MQ method appearing since 2009 [80], the first SG GCs containing LiYF_4_ crystals dates back to 2013 with the works of Kawamura et al. [81] and Secu et al. [82]. In both cases the authors used Fujihara’s synthesis, and Li and F were added in excess to avoid losses due to evaporation and guarantee LiYF_4_ formation. In fact, a stoichiometric Y:Li ratio favored the crystallization of YF_3_ instead of LiYF_4_. Gel powders were then treated between 500 and 600 °C. Kawamura et al. obtained a mix of LiYF_4_ and YF_3_ phases when the Li:Y molar ratio was lower than 3, while only pure LiYF_4_ phase was produced using this ratio. By EDXS analysis, Nd^3+^ incorporation into the fluoride NCs was proven even though observable amounts of F, Nd, and Y were also detected in the glass matrix. UC spectra were obtained when exciting the samples at 800 nm, showing crystal-like behavior. The Nd^3+^ lifetime in LiYF_4_ was longer than in YF_3_, suggesting a better Nd^3+^ distribution for the former. The same authors prepared the same materials with only LiYF_4_ crystals and treated the samples with HF to remove the SiO_2_ matrix [83]. However, relevant amounts of O and Si were detected after HF treatment and PL measurements did not show a significant difference after and before the HF treatment, thus ensuring the presence of Nd^3+^ ions in the fluoride NCs.

Secu et al. [82,84] prepared (100 − x)SiO_2_-xLiYF_4_ GC powders doped with Eu^3+^ and Er^3+^-Yb^3+^ and compared the results for LiYF_4_:Eu^3+^ and LiYF_4_:Er^3+^-Yb^3+^ crystal pellets. As compared to an untreated xerogel, Eu^3+^-doped GC samples showed 7–8-fold stronger emission, a much longer lifetime of the ^5^D_0_-^7^F_2_ red emission (613 nm), and better resolved bands due to the Stark splitting and reduced inhomogeneous broadening typical of amorphous environments. Crystal pellets and GCs showed similar PL spectra and lifetimes. For Er^3+^-Yb^3+^-doped samples, UC emission was detected in both GCs and crystal pellets. A saturation effect of the red UC emission was observed for GC samples, associated with the high Ln^3+^ ions concentration, which caused a back-ET from Er^3+^ to Yb^3+^. 

**Figure 7:**
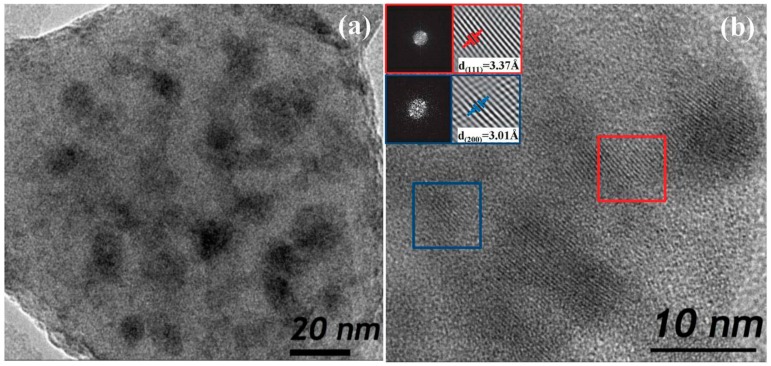
(**a**) TEM and (**b**) HRTEM images of 95SiO_2_-5BaGd_(1−x)_Eu_x_F_5_. Inset show power spectrum (FFT pattern) and filtered higher-contrasted red and blue square nanoparticles [86].

BaYF_5_ and BaGdF_5_ are other interesting crystal phases suitable for photonic applications. The first 95SiO_2_-5BaYF_5_ GC obtained by SG was prepared recently using Eu^3+^ and Sm^3+^ as dopants [85]. After heat treatment at 750 °C cubic BaYF_5_ NCs, 11 nm in size, were detected by HRTEM and XRD. For comparison, BaYF_5_ NCs were synthesized by the solvo-thermal method and dispersed in toluene. Similar PL features and lifetimes were obtained for the GC and the dispersed NCs. The same authors also prepared 95SiO_2_-5BaGd_(1−x)_RE_x_F_5_ (x = 0 or 0.02 mol %, where RE = Eu^3+^, Sm^3+^, Dy^3+^ and Tb^3+^) GC [86]. After heat treatment at 650 °C, BaGdF_5_ NCs, with a size around 10 nm, precipitated in the SiO_2_ matrix (Figure 7). Upon Gd^3+^ excitation at 272 nm or Eu^3+^ direct excitation at 393 nm, very similar Eu^3+^ emission spectra were obtained and the bands showed typical Stark components that were quite well resolved, thus confirming the incorporation of Eu^3+^ in the BaGdF_5_ NCs and the ET from Gd^3+^ to Eu^3+^.

### 1.3. Other Glass-Ceramics (Oxides and Oxyclorides)

In this section we resume work concerning GCs based on different compositions containing oxide and oxychloride NCs. Oxide GCs have received great attention because some oxide phases present interesting properties. For example, it is worth citing SnO_2_, which is a wide gap n-type semiconductor with strong UV absorption (energy gap ~3.6 eV at 300 K) and tunable emission spectra depending on the crystal size. In fact, SnO_2_ is known to act as a Quantum Dot (QD) when the crystal size is smaller than or comparable to the Bohr radius, thus showing properties between bulk semiconductor and discrete molecules that have many applications in several technological fields. Significant blue-energy shift of the intrinsic absorption edge can be obtained by strong quantum confinement of excitons inside the QDs [87,88]. The incorporation of SnO_2_ NCs in an SiO_2_ glass matrix using SG dates back to 2002 with the works of Chiodini et al. [87] and Nogami et al. [89] Chiodini et al. reported on 98SiO_2_-2SnO_2_ materials obtained by mixing TEOS and dibutyltin diacetate (Sn(CH_2_CH_2_CH_2_CH_3_)_2_(OOCCH_3_)_2_) in ethanol and using a TEOS:H_2_O molar ratio of 1:8. The samples were heated from 450 to 1050 °C in an O_2_ atmosphere and tetragonal SnO_2_ NCs with a size of 1.5–2 nm were observed by HRTEM between 950 and 1050 °C. However, the NCs’ size and distribution are strongly dependent on the atmosphere. NCs above 10 nm (clusters) were obtained for heat treatment at 1050 °C in reducing atmosphere. The near-UV absorption edge shifts to high energies by decreasing the synthesis temperature due to a decrease in the SnO_2_ NCs’ size. The author concluded that a possible application of these materials could be as all-optical switching devices. The authors also reported that an increase in the SnO_2_ content up to 15 mol % produces negative photorefractivity, activated by UV-Vis light [90]. A refractive index change of −4 and −6, measured at 980 nm, was obtained after sample irradiation at 266 and 532 nm, respectively.

Nogami et al. prepared materials with compositions (100 − x)SiO_2_-xSnO_2_ (x = 1, 3 and 5 mol %) doped with 1 mol % of Eu_2_O_3_ [89]. They used TEOS as the SiO_2_ precursor and SnCl_2_·2H_2_O together with EuCl_3_ as SnO_2_ and Eu^3+^ precursors, respectively. After a gelation period at room temperature for two weeks, bulk samples were treated from 500 to 1000 °C to ensure the densification of the glass matrix and allow SnO_2_ NCs’ precipitation. For compositions containing 3 and 5 mol % of SnO_2_, NCs with a size of 6.9 and 8.5 nm were obtained, while no NCs were observed for 99SiO_2_-1SnO_2_ composition. Absorption measurements showed a blue shift of the band-tail with respect to SnO_2_ bulk sample. Higher blue-shift and energy gap were obtained when decreasing the SnO_2_ content, related to a smaller NCs size. PL showed that Eu^3+^ emission intensity increases proportionally to the third power of SnO_2_ concentration: Eu^3+^ emission in a 95SiO_2_-5SnO_2_ sample was 150 times higher than in 99SiO_2_–1SnO_2_. PL excitation spectra revealed an ET process between Eu^3+^ and SnO_2_ NCs, thus indicating the incorporation of Eu^3+^ in the crystal phase and making possible its emission upon SnO_2_ excitation. Other authors studied (100 − x)SiO_2_-xSnO_2_ (x = 1–10 mol %) GCs doped with Eu^3+^ and Eu^3+^-Tb^3+^ following Nogami’s synthesis [91,92,93]. By increasing the SnO_2_ content up to 10 mol % (NCs size ~5 nm), a low quantum confinement effect was observed. Hence, most research was performed on GCs containing 5 mol % of SnO_2_ and the optical characterization showed that in strong confinement conditions the energy gap has a high dependence on the NCs’ size. Eu^3+^ ions incorporated in SnO_2_ NCs were excited in the range 340–394 nm and, considering the variation of the band gap with the crystal size, the use of a certain excitation wavelength allowed for exciting only NCs with a defined crystal size, thus producing remarkable variation in the PL emission spectra. ET from SnO_2_ NCs to Tb^3+^ ions was observed in Eu^3+^-Tb^3+^-doped GCs. It was observed that the ET depends on the crystal size, being favored for the smallest NCs. A relevant drawback related to SnO_2_ NCs is the low solubility of Ln^3+^ limited to ~0.05%, the remaining ions being segregated at grain boundaries, probably in the form of Ln_2_Sn_2_O_7_ crystals [94]. To overcome this limitation, Van Tran et al. [95,96] prepared SiO_2_-SnO_2_ GCs with a maximum nominal NCs concentration of 20 mol % to allow for higher Ln^3+^ amount incorporation in SnO_2_ NCs. The authors prepared Er^3+^-doped materials and PL measurements showed that an increase in SnO_2_ concentration promotes Er^3+^ ions’ incorporation in SnO_2_ NCs.

Among SiO_2_ glass matrices containing oxide NCs, those containing ZrO_2_ crystals deserve special attention because ZrO_2_ is a pretty cheap material, transparent over a wide range of wavelengths: from 300 nm to 8 μm, it has quite low phonon energy ~650 cm^−1^ and a high refractive index. Such properties have been exploited to develop Ln^3+^-activated planar waveguides at telecom wavelength [97,98]. (100 − x)SiO_2_-xZrO_2_ (x = 10–30 mol %) homogenous and crack-free thin films doped with Er^3+^ were prepared following a synthesis route similar to SnO_2_, using TEOS and ZrOCl_2_·8H_2_O as SiO_2_ and ZrO_2_ precursors, respectively. All waveguides showed the existence of one mode at 1550 nm with relatively low propagation losses; the refractive index increased from 1.492 to 1.609 for 10 and 30 mol % ZrO_2_, respectively. Narrow Er^3+^ emissions were observed in thin films with respect to glass materials, thus indicating Er^3+^ incorporation in ZrO_2_ NCs. Suhaimi et al. [99] prepared several (100 − x)SiO_2_-xZrO_2_ compositions (x = 30–70 mol %) doped with 0.58 mol % Er^3+^. The refractive index at 1550 nm changed linearly from 1.6931 to 1.7334 by increasing ZrO_2_ content. Much higher PL emission of Er^3+^ at 568 nm was observed for films containing 70 mol % of ZrO_2_, thus indicating that ZrO_2_ facilitates Er^3+^ to disperse homogeneously and the low phonon energy of the crystal phase reduces non-radiative losses. Other authors also studied SiO_2_-ZrO_2_ waveguides doped with Er^3+^-Yb^3+^ with ZrO_2_ contents up to a maximum of 25 mol % [100]. Low roughness, a crack-free surface, and a high confinement coefficient were observed for all the compositions. Er^3+^ NIR PL was enhanced when the waveguide was co-doped with Yb^3+^, denoting an efficient ET between these ions. The authors considered the possibility of applying these materials as EDWA and WDM. Many GCs containing other oxide NCs have also been developed as waveguides [101,102,103,104].

To conclude this section, we mention the work of Secu et al. on oxychloride GCs [105]. 95SiO_2_-5LaOCl GCs doped with 1 mol % Eu^3+^ were prepared following a similar synthesis to LaF_3_ GCs but replacing TFA with trichloacetic acid (CCl_3_COOH). LaOCl:Er^3+^ pellets have also been prepared using a conventional solid state reaction between lanthanum oxide and ammonium chloride. DTA analysis showed that several exothermic peaks appear for measurements performed in air, but these peaks disappear completely for measurements in Ar. However, XRD confirmed the formation of LaOCl NCs, 20–60 nm in size, during heat treatment at 450–750 °C in air. For heat treatment in Ar, smaller crystal size and fraction were obtained. Judd–Ofelt analysis along with PL measurements showed that, as the annealing temperature increases, a higher amount of Eu^3+^ ions is incorporated in LaOCl NCs, thus producing a better resolved Stark component and a much longer lifetime with respect to xerogel. Similar results were obtained for LaOCl:Eu^3+^ pellets, indicating the effective incorporation of Eu^3+^ in this crystal phase. 

### 1.4. Prospects and Perspectives

In summary, most of the transparent SG OxGCs previously described are based on a unique synthesis developed by Fujihara et. al., using TMOS and/or TEOS as the SiO_2_ precursor, TFA as the fluorine source, and with an upper limit of nominal crystal phase around 10 mol %. The real crystal fraction in GCs was never estimated by a reliable method as Rietveld refinement and the few works that reported such information only used optical results to estimate the crystal content. Furthermore, many papers describe materials with 5 mol % of nominal active phase, this being even less than that achievable by classical MQ and requiring an extremely long time for bulk sample preparation, from several weeks up to months. Moreover, quite high treatment temperatures, up to 1000 °C, are used to crystallize the fluoride NCs when the crystallization of fluorides detected by DTA occurs at around 300 °C. For all these reasons, research in the SG OxGCs field shows good and enhanced optical properties but no improvement of the synthesis, and no new processing methods have been reported in the literature since the first papers published 20 years ago. 

In recent years, the GlaSS group of CSIC has been working on the optimization of the synthesis for significantly increasing the crystal content of SG OxGCs. In fact, it is worth noting that synthesis parameters such as molar ratios between precursors, temperature, and time of reaction are strictly dependent on the crystal phases and their content. Moreover, a unique synthesis, in general, is not suitable to obtain different compositions for the same crystal phase, and modifications of the synthesis are necessary to obtain novel materials. In addition, we partially replaced TEOS with methyltriethoxysilane (MTES) in the SiO_2_ sol synthesis, reducing the sintering temperature to 550 °C and making it possible to obtain enhanced optical properties without a need for much higher treatment temperatures. 

## 2. Experimental

### 2.1. Synthesis of (100 − x)SiO_2_-xLaF_3_

Our research started with LaF_3_ GC films prepared by dip-coating. The precursors used were TEOS, H_2_O (0.1 M HCl), EtOH, TFA, and La(CH_3_COO)_3_. Dopants were added as acetates. A first SiO_2_ sol was prepared using a molar ratio 1TEOS:2H_2_O (0.1 HCl):9.5EtOH stirred for 2 h at room temperature. Separately, 1La(CH_3_COO)_3_:5EtOH:5TFA:4H_2_O were mixed and stirred for 2 h at 40 °C in a glycerine bath. By mixing different volumes of SiO_2_ sol with the La solution, 90SiO_2_-10LaF_3_ and 80SiO_2_-20LaF_3_ compositions were obtained. To further increase the nominal LaF_3_ concentration to 30 mol %, the La solution was modified, adding more TFA (7 mol %) to favor acetate dissolution. The last composition we obtained is 60SiO_2_–40LaF_3_ and in this case a further increase of TFA up to 10 mol % was necessary to obtain transparent thin films. Thin films were deposited by dip-coating on silica or Si substrates using a withdrawal rate of 20–45 cm/min. The thickness and refractive index of thin films were measured by a 2000 U ellipsometer (J.A. Woollam Co., Inc., Lincoln, NE, USA), using a Cauchy dispersion relation as a model.

Self-supported layers (or bulk-like samples) were also prepared with compositions 90SiO_2_-10LaF_3_ and 80SiO_2_-20LaF_3_ using the modified ratio of SiO_2_ sol 1TEOS:7.5H_2_O (0.1 HCl):5EtOH while leaving unchanged the synthesis of La solution described before for thin films with the same composition. Samples were dried at 50 °C over two days and then the covering was removed, letting the solvent evaporate for seven days. 

A further modification to obtain higher thickness of thin films and improve the mechanical resistance of self-supported layers was to partially replace TEOS with MTES in the ratio 40TEOS:60MTES [106]. A modification of the molar ratios was necessary for thin film preparation to obtain the highest film thickness along with good sol stability. In particular, for thin films the SiO_2_ sol was prepared using 0.4TEOS:0.6MTES:1H_2_O (0.1 HCl):2.5EtOH:0.2CH_3_COOH, while the La solution was left unchanged. For self-supported layers, the previous TEOS:H_2_O ratio was used, replacing TEOS with 0.4 TEOS + 0.6 MTES (mol %) to maintain the same ratio of SiO_2_ precursor and H_2_O, which is a crucial parameter for bulk-like samples. 

### 2.2. Synthesis of (100 − x)SiO_2_−xGdF_3_/NaGdF_4_

90SiO_2_-10GdF_3_ and 80SiO_2_-20LaF_3_ self-supported layers were also obtained following the two-step process and using the aforementioned TEOS:MTES ratio for SiO_2_ sol. In this case Gd solution, using Gd(CH_3_COO)_3_ as precursor, was stirred for 24 h at 40 °C to obtain a homogeneous solution of the products, thus making possible the crystallization of GdF_3_.

Finally, we prepared for the first time SiO_2_-NaGdF_4_ materials as self-supported layers and thin films. A SiO_2_ sol was prepared using only TEOS as the SiO_2_ precursor with the same ratios used for SiO_2_-LaF_3_ compositions. Then, Na(CH_3_COO), Gd(CH_3_COO)_3_, EtOH, TFA, and H_2_O were mixed using similar ratios of TFA and EtOH and trying several Na:Gd ratios (1.125–0.80):1. The solution was stirred 24 h at 40 °C in a glycerin bath. 

For all compositions, GCs were obtained after heat treatment at 550–750 °C from 1 min up to several hours using heating rates of 1–10 °C/min. In all cases, thermal quenching in air was performed to obtain good crystallization of the samples. 

### 2.3. Thermal and Structural Characterization

DTA was performed in air and argon (inert) atmosphere on small pieces of 1–1.25 mm in size, with heating rates 10–40 °C using SDT Q600 (TA Instruments, New Castle, DE, USA) equipment. Measurements were performed in the range 25–800 °C using 15–30 mg of sample.

High-resolution XRD patterns were collected in the range 6–27° at the synchrotron SpLine BM25B of the ESRF (European Synchrotron Radiation Facility, Grenoble, France) using a wavelength of 0.619 Å and a step size of 0.02°.

HRTEM was performed using a JEOL 2100 microscope (Akishima, Tokyo, Japan). Samples were prepared using lacey formvar carbon film that had a small amount of scratched sample deposited on them. 

Fourier Transform Infra-Red (FTIR) spectra in the range 2000–600 cm^−1^ were obtained, with a resolution of 4 cm^−1^, using a Perkin Elmer Spectrum 100 instrument (Waltham, MA, USA).

^19^F magic-angle spinning nuclear magnetic resonance (^19^F MAS/NMR) spectra of xerogel and GCs were recorded using a NMR Spectrometer AVANCE II (BRUKER, Billerica, MA, USA) equipped with a 9.4 Tesla magnet (400 MHz) and a 2.5 mm rotor spinning at 20 kHz.

X-ray Absorption Spectroscopy (XAS) was measured at the SpLine BM25A of the ESRF collecting the spectra in fluorescence mode using a 13-element Si (Li) solid-state detector with the sample surface placed at an angle of 45° to the incident beam. Six scans were acquired to obtain an average spectrum. XAS data were processed using ATHENA software [107]. Eu_2_O_3_ and EuF_3_ crystal powders were also measured as reference materials to compare their spectra with those of Eu^3+^-doped xerogel and GC samples. 

Photoluminescence measurements were recorded using a FS5 fluorescence spectrometer (Edinburgh Instruments Ltd., Livingston, UK) equipped with a 150 W Xenon lamp or Ti:sapphire ring laser (0.4 cm^−1^ linewidth) in the 770–920 nm spectral range. The emission was detected by Hamamatsu H10330A-75 or Hamamatsu R928P photomultiplier (Hamamatsu City, Shizuoka, Japan). 

Even though several compositions were prepared, most of the results shown in the next sections deal with 80SiO_2_-20LaF_3_/GdF_3_/NaGdF_4_ compositions, which have been studied in detail in recent years, all containing 20% of fluoride phases, at least double that reported by other authors.

## 3. Results and Discussion

### 3.1. Materials

Transparent and crack-free GC thin films were obtained for all compositions, even for those containing 30 or 40 mol % of active phase. For single deposition at 30 cm/min and heat treatment at 550 °C for 1 h, thicknesses around 500 nm and 1 μm were obtained for TEOS and TEOS/MTES compositions, respectively. Higher thicknesses up to 900 nm and 1.7 μm were obtained for TEOS and TEOS/MTES, respectively, depositing two films or increasing the withdrawal rate. Hence, the addition of MTES in the SiO_2_ sol allows a notable increase of the thickness, thus making this precursor attractive for application where a thin film of 1 μm or thicker is required. Transparent and high-quality films were obtained, as confirmed by a good agreement between ellipsometry measurements and fits. 

Transparent and crack-free self-supported layers were also obtained after heat treatments. The addition of MTES allows increasing the heating rate up to 10 °C/min without the appearance of cracks. 

### 3.2. Thermal and Structural Characterization 

#### 3.2.1. SiO_2_–LaF_3_

**Figure 8:**
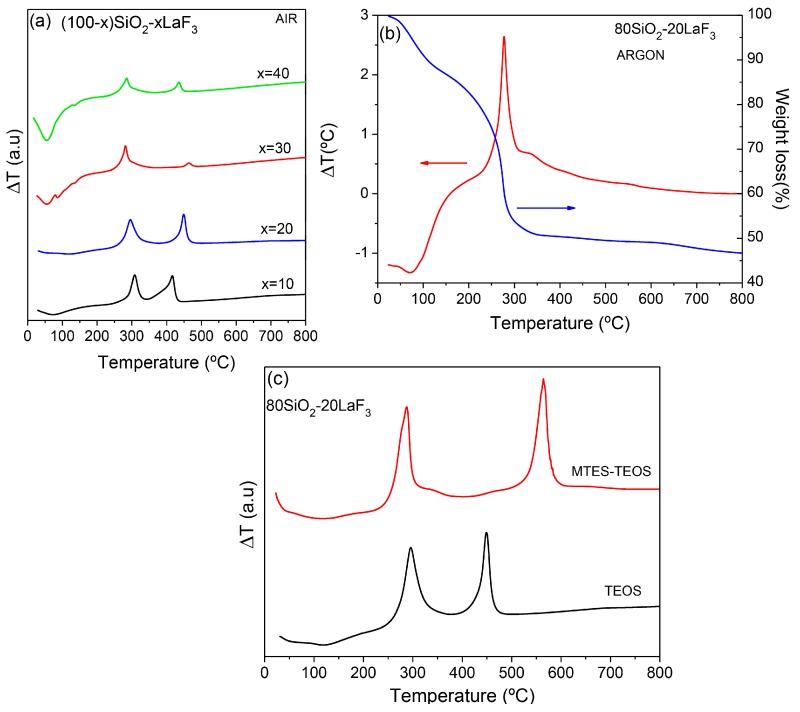
(**a**) DTA in air of (100 − x)SiO_2_-xLaF_3_ (x = 10–40 mol %) bulk samples prepared with TEOS; (**b**) DTA and TG in argon atmosphere of 80SiO_2_-20LaF_3_ bulk samples prepared with TEOS; (**c**) DTA in air of 80SiO_2_-20LaF_3_ bulk samples with TEOS and TEOS/MTES. All measurements were performed using a heating rate of 10 °C/min.

DTA measurements for (100 − x)SiO_2_-xLaF_3_ (x = 10–40 mol %) bulk samples obtained drying thin films sols are shown in Figure 8a. The curves show a first endothermic peak with a weight loss (not shown) associated with H_2_O and other solvent removal. Such an endothermic process is much more intense for a composition with a higher amount of La. This could be associated with the increasing water content passing from x = 10 to x = 40. 

Then, a first exothermic peak appears around 300 °C, together with a big mass loss that increases passing from x = 10 to x = 40 mol %. The peak shifts towards lower temperatures when increasing the active phase content. As reported by other authors [43], such a peak is associated with the crystallization of LaF_3_, favored by increasing the La content. Such crystallization takes place after a chemical decomposition with gas release, which is responsible for the notable weight loss. The intensity of the endothermic and exothermic peaks is affected by the sample amount used for the analysis, and therefore it cannot be compared quantitatively. The second exothermic peak in the range 400–500 °C is assigned to organic combustion and was not detected in an argon atmosphere (Figure 8b). The effect of the SiO_2_ precursor on the DTA curve is shown in Figure 8c for 80SiO_2_-20LaF_3_ samples prepared using TEOS and TEOS/MTES in the ratio 40:60 as SiO_2_ precursor. The crystallization peak appears centered around the same value, near 300 °C, while the second exothermic peak is associated with organic combustion of different species. In fact, when MTES is used, CH_3_ groups are introduced in the system and released around 550–600 °C. Such release is associated with a contraction of thin films and self-supported layers. Therefore, when MTES is used, treatment temperatures no higher than 550 °C should be used to avoid sample cracking. However, and concerning the crystallization mechanism, no relevant effects are introduced by the partial replacement of TEOS with MTES. The introduction of MTES improved the mechanical resistance during the treatment process and MTES bulk-like samples can be treated using a 10 °C/min heating rate instead of 1–2 °C/min, as is commonly used for samples prepared using only TEOS. Moreover, MTES addition facilitates –OH group removal [108], without requiring extremely high treatment temperatures (900–1000 °C).

**Figure 9:**
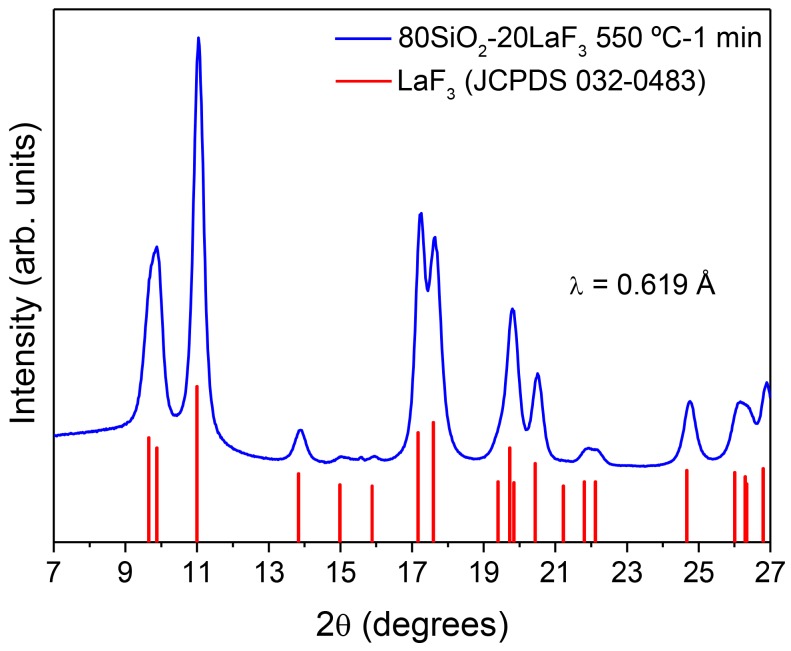
XRD of 80SiO_2_-20LaF_3_ GC treated at 550 °C for 1 min performed at the synchrotron SpLine BM25B of the ESRF.

The XRD of 80SiO_2_-20LaF_3_ bulk sample prepared using TEOS/MTES and treated at 550 °C for 1 min is represented in Figure 9. Well-defined diffraction peaks are observed even for this fast treatment; the crystal size, estimated by Scherrer’s equation, is around 8.5 nm. No relevant SiO_2_ amorphous pattern is observed, different to the XRD results obtained by other authors [50,51,52,53,54,55,57], indicating a relevant increase of fluoride crystal fraction. Dopant incorporation, such as Er^3+^, was confirmed by an appreciable shift of the diffraction peaks towards higher angles, associated with a shrinking of the unit cell due to the lower size of Er^3+^ with respect to La^3+^ [109]. By Rietveld refinement (not shown), we obtained a LaF_3_ crystal fraction of 18 wt %, which is, to the best of our knowledge, the highest value of fluoride concentration ever reported in OxGCs. The crystallization mechanism of LaF_3_ in SG OxGCs has been studied in a previous paper [91]. It was shown that the LaF_3_ crystallization mechanism is very different to that for MQ GCs. In fact, for MQ GC a diffusion-controlled process is responsible for the crystallization of LaF_3_ and many other fluoride phases; phase separation acts as a precursor for crystallization. On the contrary, LaF_3_ crystallization in SG OxGCs is not a diffusion-controlled process but consists of a fast crystal precipitation taking place after a chemical decomposition. It was observed that LaF_3_ NCs are not stable for heat treatment at crystallization or higher temperatures and amorphization was observed for a long treatment time (5–80 h) at 550 °C. 

**Figure 10:**
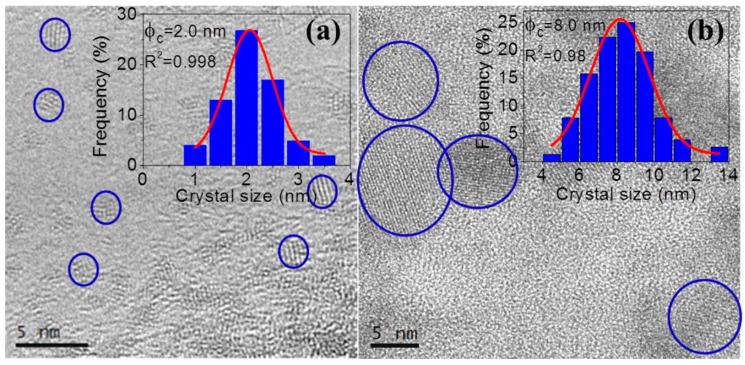
HRTEM of 80SiO_2_-20LaF_3_ (**a**) thin film and (**b**) self-supported layer prepared using TEOS/MTES and treated at 550 °C for 1 min. The corresponding crystal size distributions are also shown.

HRTEM micrographs of 80SiO_2_-20LaF_3_ thin films and self-supported layers treated at 550 °C for 1 min are shown in Figure 10a,b, respectively. Very small LaF_3_ NCs around 2–3 nm are observed in thin film samples, as confirmed by the crystal size distribution shown in the inset. For self-supported layers the crystal size is much bigger, around 8 nm, in agreement with the XRD results in Figure 3. In both cases homogeneously distributed NCs are observed, without the formation of clusters, even for such a fast heat treatment at 550 °C for 1 min.

**Figure 11:**
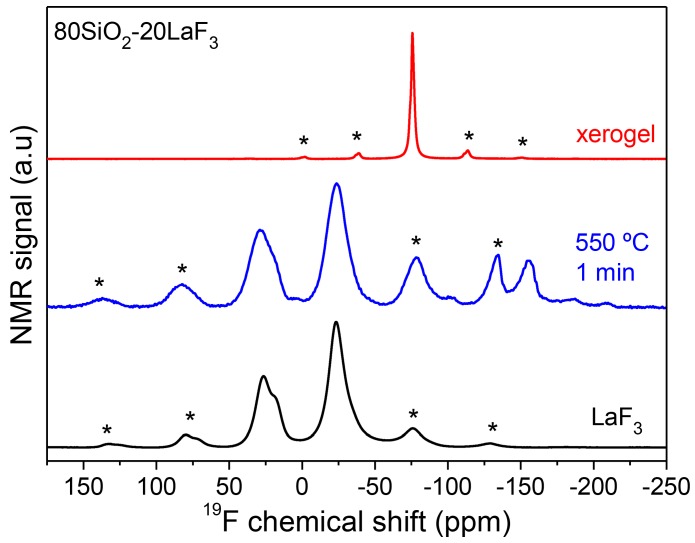
^19^F MAS-NMR spectra of 80SiO_2_-20LaF_3_ xerogel and GC treated at 550 °C for 1 min. The spectrum of pure LaF_3_ crystal powder is also given for comparison. Stars indicate spinning sidebands.

NMR spectra of SG OxGCs are rarely encountered in the literature but the information that can be extracted is of relevant importance. In particular, ^19^F NMR spectra allow for obtaining evidence about fluorine surrounding in the xerogel and how it changes after the crystallization process. Figure 11 shows ^19^F MAS NMR of 80SiO_2_-20LaF_3_ xerogel and GC treated at 550 °C for 1 min [110]. The spectrum of polycrystalline LaF_3_ was also acquired for comparison. In the xerogel sample the fluorine surrounding is as in the precursor TFA acid and no bonding with Si is observed. After heat treatment at 550 °C for 1 min, the spectrum of the GC sample is practically the same as that of the LaF_3_ polycrystalline powders. This further indicates that a chemical reaction accompanied by fast crystal precipitation is responsible for the LaF_3_ NCs’ formation.

**Figure 12:**
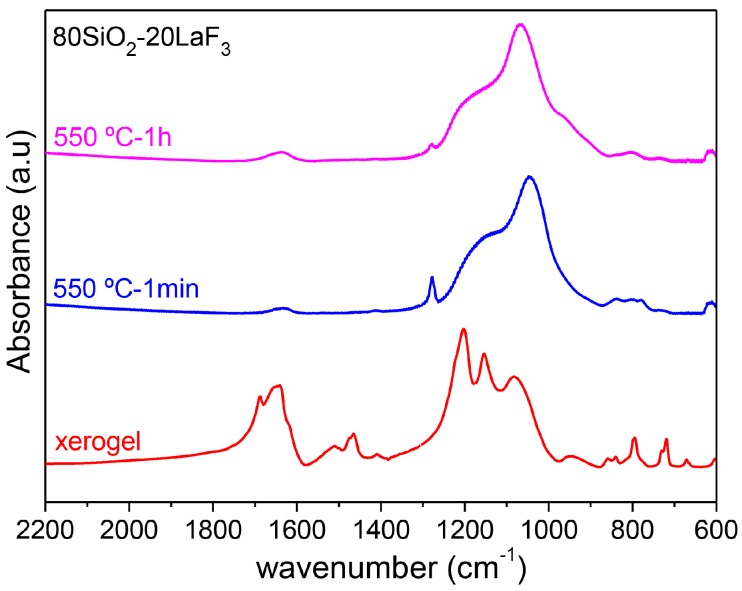
FTIR of 80SiO_2_-20LaF_3_ xerogel and GC self-supported layer treated at 550 °C for 1 min and 1 h.

These results are further confirmed by FTIR spectra of 80SiO_2_-20LaF_3_ xerogel and GCs treated at 550 °C for 1 min and 1 h, as shown in Figure 12 [110]. In the xerogel, the bands centered at 1680 and 1650 cm^−1^ are associated with H-O-H bending and C=O stretching vibrations, respectively. In the range 1500–1400 cm^−1^ several absorption bands appear and are assigned to TFA, acetates, and/or derived ions [43]. A C-F stretching band is also identified between 1400 and 1000 cm^−1^. All of these bands disappear in the GC sample, accompanied by LaF_3_ NCs’ precipitation. In the GC the bands in the range 1100–800 cm^−1^ are associated with Si-O-Si asymmetric and symmetric stretching vibrations of [SiO_4_] units. A small band at 1279 cm^−1^, also present in the xerogel sample but much more intense, is assigned to C-O vibration and due to incompletely removed organic compounds. Such a band disappears over longer treatment times at 550 °C.

**Figure 13:**
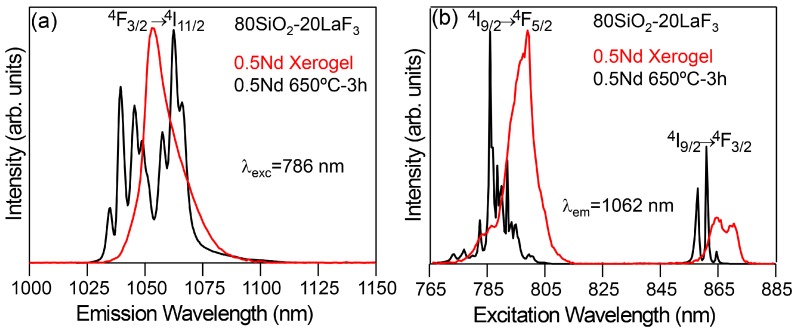
PL (**a**) emission and (**b**) excitation spectra of 80SiO_2_-20LaF_3_ bulk xerogel and GC treated at 650 °C for 3 h. All spectra were recorded at 9 K.

Photoluminescence (PL) measurements are of great importance in most papers about SG OxGCs; in particular, enhanced properties are obtained when dopants are embedded into the fluoride NCs with low phonon energy. As an example, low-temperature (9 K) PL emission and excitation spectra of 80SiO_2_-20LaF_3_ xerogel and GC treated at 650 °C for 3 h and doped with 0.5 Nd^3+^ are shown in Figure 13 [111]. Both the emission and excitation spectra of the xerogel show broad and less structured bands, indicating a predominant amorphous environment for Nd^3+^ ions. Instead, for the GC sample sharp peaks and well-resolved Stark components are observed and associated with Nd^3+^ emission in LaF_3_ NCs. Similar features are observed for the excitation spectrum, where well-resolved peaks are observed for the ^4^I_9/2_→^4^F_5/2_ band; moreover, the ^4^I_9/2_→^4^F_3/2_ doublet narrows and splits into two main single components, as expected for a well-defined crystal field site. Therefore, Nd^3+^ incorporation into LaF_3_ NCs was unambiguously confirmed.

#### 3.2.2. SiO_2_–GdF_3_/NaGdF_4_

**Figure 14:**
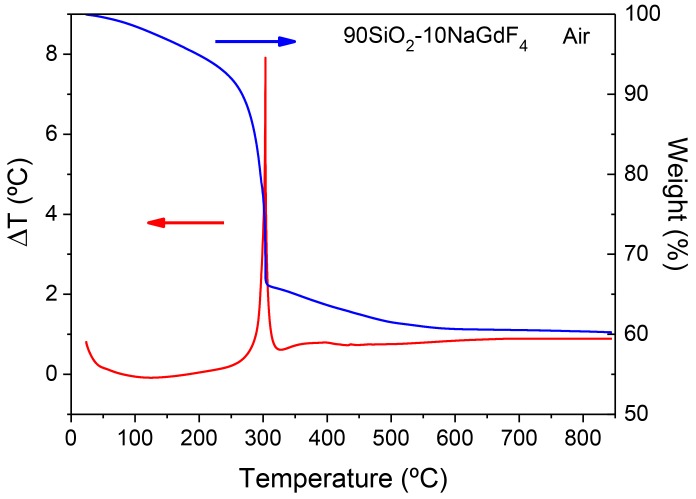
DTA (red) and TG (blue) curve of 90SiO_2_-10NaGdF_4_ bulk sample acquired in air using a heating rate of 10 °C/min.

Figure 14 shows DTA curves of the 90SiO_2_-10NaGdF_4_ bulk-like sample. The weight loss between 70 and 200 °C, of less than 10%, is ascribed to solvent removal. Then, as usually occurs for SG oxyfluoride compositions, a strong and sharp exothermic peak appears around 300 °C along with a mass loss of 30%. Such an exothermic peak is associated with chemical decomposition with NaGdF_4_ precipitation, similar to that described by other authors for other fluoride crystal phases [43,57,82]. Further weight loss for heat treatment in the range 400–600 °C can be associated with the combustion of organic compounds. The same sample measured in an argon atmosphere showed similar characteristics; therefore, the exothermic peak is associated with NaGdF_4_ crystallization. Similar features are obtained for SiO_2_-GdF_3_ composition and described elsewhere [112].

**Figure 15:**
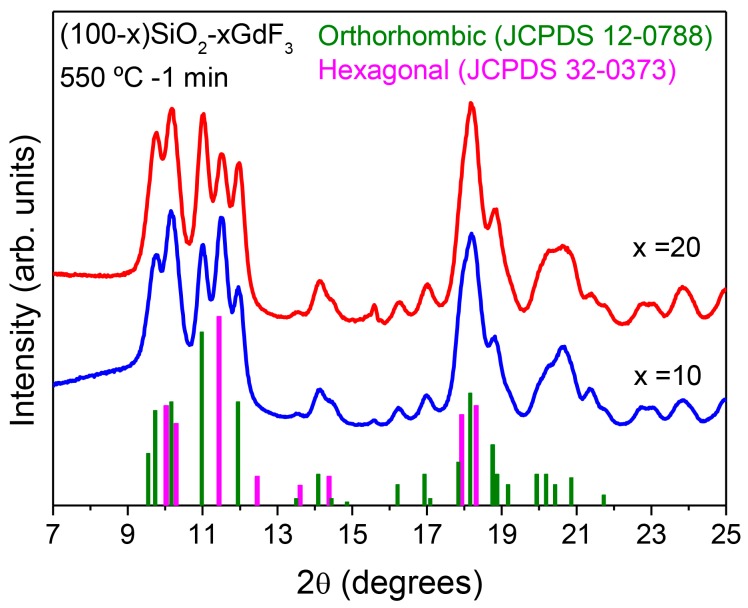
XRD of (100 − x)SiO_2_-xGdF_3_ (x = 10 and 20 mol %) GC treated at 550 °C for 1 min performed at the synchrotron SpLine BM25 of the ESRF.

Diffractograms of (100 − x)SiO_2_-xGdF_3_ (x = 10 and 20 mol %) treated at 550 °C for 1 min are shown in Figure 15. As observed, both phases of GdF_3_, orthorhombic and hexagonal, appear even for such fast heat treatment. It was shown that from LaF_3_ to EuF_3_, the hexagonal phase is preferred, while elements heavier than Gd are organized in orthorhombic structures [113]. Considering that Gd is right in the middle of a lanthanide series, it seems reasonable that a mixture of both phases appear. The relative intensity of hexagonal and orthorhombic structures is quite similar for all heat treatments, thus suggesting that there is no one preferential crystal structure but that both configurations coexist. Crystal size is around 8 and 9 nm for hexagonal and orthorhombic phases, respectively. Small changes in relative peak intensities can be associated with the deformation or preferential incorporation of Eu^3+^ ions at certain crystallographic sites. Moreover, the fact that similar sizes are obtained for both compositions suggests that NC formation is not related to the amount of the initial precursors, the mechanism being explained as fast crystal precipitation after a chemical reaction similar to that described by Fujihara for LaF_3_ [43]. However, the final crystal fraction should be affected by the initial content as observed for LaF_3_ compositions. Nevertheless, for GdF_3_ crystallization a quite different scenario is observed with respect to LaF_3_ crystallization, where an amorphization was observed over a long heat treatment time at 550 °C. In fact, no changes in the crystal size are observed for treatment times up to 8 h; the diffractograms of GCs treated for 1 min up to 8 h being practically the same. It seems that GdF_3_ NCs are more stable against decomposition as opposed to LaF_3_ ones. Further work is still necessary to clarify this point. 

It is also worth noting that SiO_2_ precursors affect the final crystal phase and the crystal size. For example, for 80SiO_2_-20GdF_3_ samples prepared using TEOS, only hexagonal crystals are detected but it was necessary to raise the temperature to 750 °C to obtain GdF_3_ crystals. Instead, for TEOS/MTES samples, even for treatment temperature as low as 550 °C for 1 min, a good crystallization was obtained but in this case both a hexagonal and an orthorhombic phase appeared. Therefore, as was shown, the synthesis precursor and the different ratios between them can strongly affect the final crystal size, crystal fraction, and symmetry of the crystal phase. However, the influence of the SiO_2_ precursors and the synthesis route on the crystallization tendency of the systems has not yet been totally elucidated.

**Figure 16:**
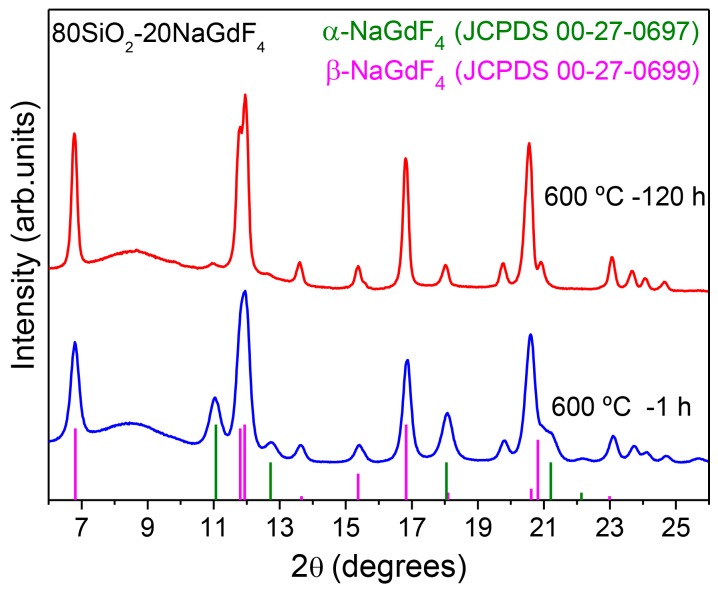
XRD of 80SiO_2_-20NaGdF_4_ GC treated at 600 °C for 1 and 120 h. The measurements were performed at the synchrotron SpLine BM25 of the ESRF.

XRD of 80SiO_2_-20NaGdF_4_ are shown in Figure 16. This is the first time that NaGdF_4_ crystals have been obtained in SG OxGCs. Diffractograms obtained for super-stoichiometric Na:Gd ratios caused the crystallization of silicates, along with NaGdF_4_. Therefore, precise control of the Na:Gd ratio was necessary to avoid the formation of silicates during heat treatment and obtain only the precipitation of fluoride NCs. It is known that Na^+^ acts as a network modifier, producing more open glass structures. In fact, Na^+^ ions can break SiO_4_ units, thus producing non-bridging oxygens with subsequent softening of the glass network. Hence, a sub-stoichiometric ratio Na:Gd 0.95:1 was necessary to ensure the complete reaction of Na with Gd, avoiding the presence of free Na^+^ ions. As observed in Figure 16, both α and β-NaGdF_4_ phases precipitated upon heat treatment at 600 °C for 1 h with a size of 4 and 13 nm, respectively. However, for longer heat treatments, up to 120 h, a relative decrease of the α phase, indicated by stars, is observed with respect to GC treated for only 1 h. Moreover, sharper peaks are observed after increasing the treatment time, thus indicating the formation of bigger crystals (more than 30 nm in size). Such behavior is quite different to that described for LaF_3_, for which an increase of the treatment time increases neither the crystal size nor the crystal fraction. Therefore, it was suggested that LaF_3_ crystals formation occurs as a fast precipitation when certain energy is given to the system, instead being a diffusion-limited process, as proposed by other authors. However, for NaGdF_4_ OxGCs, a different crystallization mechanism occurs for polymorphous crystals; in particular, the addition of alkaline earth elements could be responsible for the better crystallinity obtained by increasing the treatment time. In fact, the time-dependent diffusion of Na^+^ ions could be relevant to achieve better crystallization. 

Bartha et al. [114] studied NaYF_4_ phosphors and observed that for heat treatment at 300 °C, both cubic and hexagonal NaYF_4_ micro crystals appeared. However, by increasing the treatment temperature to 400–600 °C, only a pure hexagonal NaYF_4_ phase was observed. The authors explained such behavior as an autocatalytic process where the initial cubic NaYF_4_ phase played a catalytic role, causing its fast self-accelerated crystallization. The energy resulting from the disintegration process of the initial NaYF_4_ crystals contributed to the growth of hexagonal NaYF_4_ phase. This mechanism could also explain the crystallization of NaGdF_4_ NCs in OxGCs but further investigation has still to be done. 

**Figure 17:**
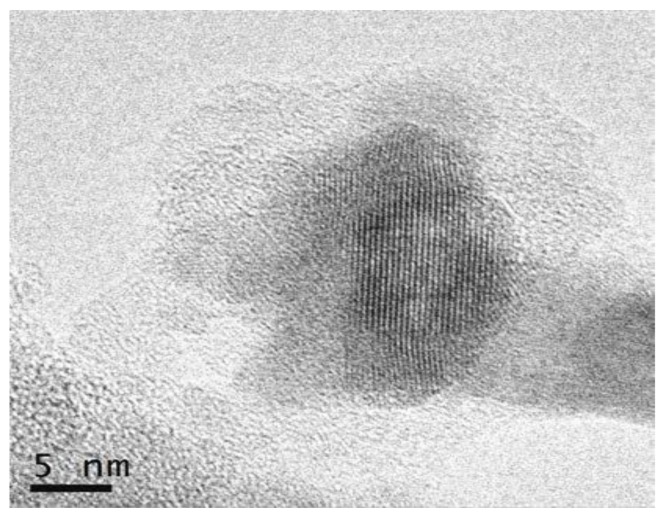
HRTEM of 80SiO_2_-20GdF_3_ self-supported layer treated at 550 °C for 1 min.

Figure 17 shows a micrograph of 80SiO_2_-20GdF_3_ self-supported layer doped with 0.5 Eu^3+^ treated at 550 °C for 1 min. As for SiO_2_-LaF_3_ GCs, homogeneously distributed NCs are observed and no agglomerates or clusters are observed, as suggested by other authors for YF_3_ NCs [57]. By a detailed analysis of the microstructures, both hexagonal and orthorhombic crystals were detected according to JCPDS. EDXS of Eu^3+^ doped 80SiO_2_-20GdF_3_ GC treated at 550 °C for 1 min revealed that Eu^3+^ ions are mostly concentrated into GdF_3_ NCs. Therefore, dopant incorporation is very fast because even for a heat treatment as short as 1 min, most Eu^3+^ ions are already embedded in the crystal phase. A better explanation of dopant incorporation is given later when the results about XAS will be discussed.

**Figure 18:**
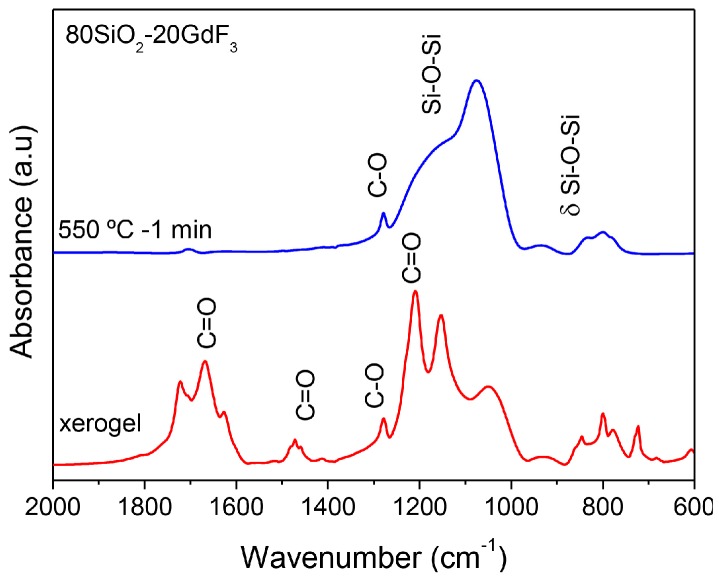
FTIR of 80SiO_2_-20GdF_3_ xerogel and GC treated at 550 °C for 1 min.

FTIR spectra of 80SiO_2_-20GdF_3_ xerogel and GC treated at 550 °C for 1 min are shown in Figure 18. Similar results are obtained with respect to the 80SiO_2_-20LaF_3_ composition given in Figure 12. For the xerogel sample TFA and acetate vibration bands are still present but disappear after heat treatment accompanied by LaF_3_ crystallization. Similar features are obtained for SiO_2_-NaGdF_4_ compositions, thus indicating that a chemical reaction followed by NC precipitation is a common feature of all the studied compositions. However, differences in crystallization behavior are also observed by XRD and therefore the crystal growth can be dependent on crystal phase and synthesis conditions. 

**Figure 19:**
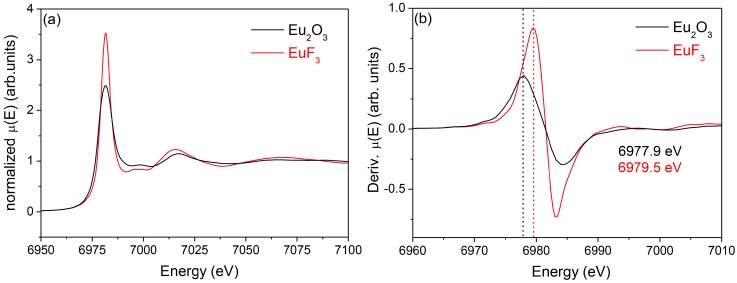
XAS (**a**) spectra and (**b**) derivate of Eu_2_O_3_ and EuF_3_ reference samples. The values shown in (**b**) refer to the maximum of the derivate curves.

**Figure 20:**
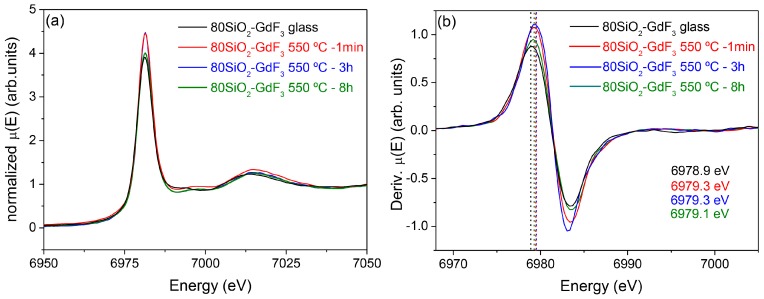
XAS (**a**) spectra and (**b**) derivate of 0.5Eu^3+^-doped 80SiO_2_-20LaF_3_ xerogel and GC samples treated at 550 °C for 1 min up to 8 h. The values shown in (**b**) refer to the maximum of the derivate curves.

The last results that are shown in this paper deal with XAS spectra of 80SiO_2_-20GdF_3_ materials doped with Eu^3+^. In particular, the spectra of the xerogel and GC sample are compared with the aim of gaining more insight into the Eu^3+^ environment. Indeed, better optical efficiencies are obtained when dopants are embedded into the NCs and knowledge of the real dopants fraction incorporated into the NCs is crucial to improve and optimize the luminescence emission of these materials. Even though a certain nominal dopant concentration is used, the real concentration into the fluoride NCs is rarely estimated, the only results being extrapolated from optical measurements [54]. Moreover, to estimate the effective concentration into the NCs, even with 10–20% error, knowledge of the crystal fraction is necessary and such values are seldom or never reported for SG OxGCs. Last year our group started acquiring data to obtain this relevant information, but a lot of work has still to be done. However, some interesting conclusions can already be drawn from the results shown below. Eu_2_O_3_ and EuF_3_ spectra are shown in Figure 19a, together with their derivate curves in Figure 19b. The maximum of the derivate curves is 6977.9 and 6979.5 eV, for Eu_2_O_3_ and ErF_3_, respectively. A lower energy is associated with less electronegativity and a lower field strength of the Ln^3+^ ion [115]. The uncertainty of the energy values is around 0.8 eV. Therefore, it can be confirmed that the two maxima are well separated from each other. Figure 20a,b show the results for a 80SiO_2_-20GdF_3_ xerogel and GC samples treated at 550 °C for 1 min up to 8 h. In all cases, the absorption maximum is centered at ~6979 eV, practically the same value obtained for EuF_3_ reference. Hence, even though by these measurements it is not possible to distinguish between a crystalline or amorphous environment, the crucial point is that a fluorine-rich environment is observed for all GCs and even for the xerogel sample. Such behavior could be explained considering that Eu^3+^ ions are still coordinated to the surrounding TFA ions in the xerogel sample, and, after heat treatment, GdF_3_ crystals precipitate together with Eu^3+^ incorporation. These results are in agreement with Eu^3+^-rich GdF_3_ NCs observed by EDXS for 80SiO_2_-20GdF_3_ GC treated at 550 °C for 1 min. 

Previous calculations performed for 80SiO_2_-20LaF_3_ samples doped with 0.5 Er^3+^ showed that almost 91% of Er^3+^ ions in GC samples are in a fluorine-rich environment, the effective concentration into the LaF_3_ NCs thus being almost one order of magnitude higher than the nominal one. Similar results were obtained for MQ samples containing LaF_3_ NCs and confirmed by PL results [116].

**Figure 21:**
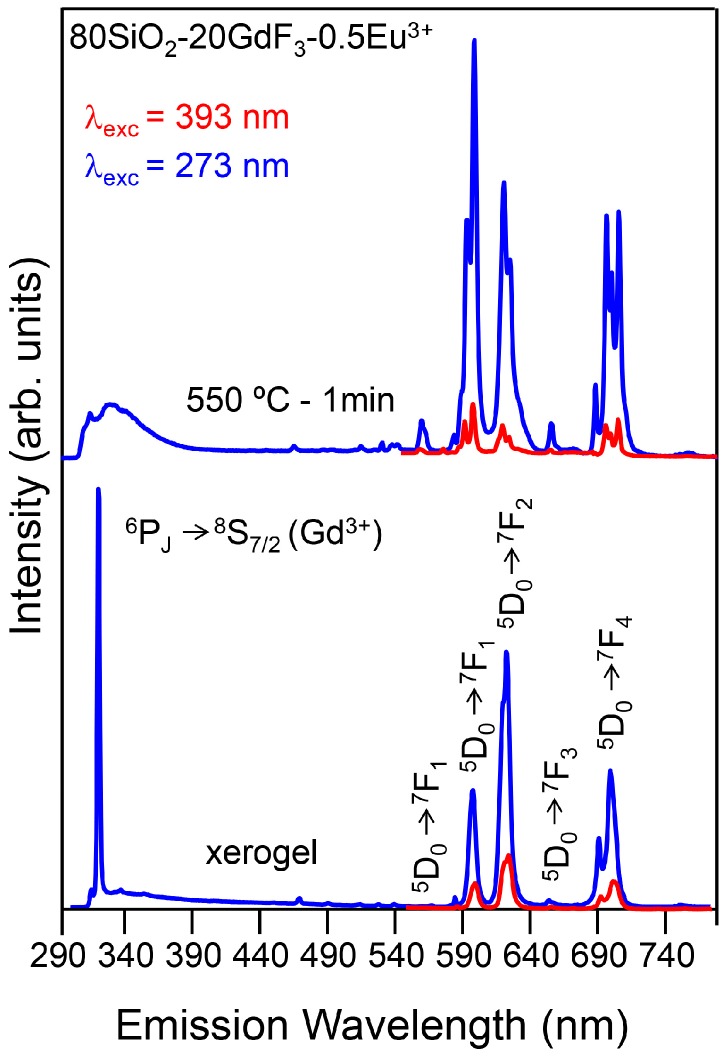
Emission spectra of Eu^3+^ and Gd^3+^ ions in xerogel (**bottom**) and GC (**top**) treated at 550 °C-1 min under excitation of Eu^3+^ at 393 nm (red spectra) and of Gd^3+^ at 273 nm (blue spectra).

Finally, to conclude this section we show some optical results for 80SiO_2_-20GdF_3_ self-supported layers doped with 0.5 Eu^3+^. Photoluminescence measurements of GC treated at 550 °C for 1 min showed well-resolved structure together with a narrowing of the Eu^3+^ with respect to the xerogel sample (Figure 21). Moreover, the *R* asymmetry ratio between the electric dipole transition (^5^D_0_-^7^F_2_) and the magnetic dipole transition (^5^D_0_-^7^F_1_) is reduced in GCs, thus indicating that Eu^3+^ ions are incorporated in the GdF_3_ crystal phases. Moreover, the ET from Gd^3+^ to Eu^3+^ was observed in the GC sample and further supported by fluorescence decay lifetimes.

## 4. Conclusions 

Different research groups have studied the preparation of transparent GC materials using the MQ process and suitable control of the synthesis and heat treatment conditions. However, SG appears as a promising alternative to obtain innovative GCs with high fluorine content and high homogeneity at a lower temperature. 

Most of the materials studied by SG are related with compositions (100−x)SiO_2_-x M_1_F_2_/M_1_F_3_/M_1_M_2_F_4_/M_1_M_2_F_5,_ containing a small nominal crystal fractions (x = 5–10 mol %), and are obtained using a similar synthesis procedure developed 20 years ago. Furthermore, the same SiO_2_ precursor, TEOS or TMOS, is used in most works, and the precursor ratio rarely changes with respect to the first papers. 

There is scarce information about the structural characterization of bulk and thin films; typical characterization is performed by means of XRD, HRTEM, and FTIR. However, most of the authors never calculated the real crystal fraction. Just one or two papers estimated the real active crystal content and concluded that for a nominal composition of 5 mol % of active phase, a 3 wt % of crystal phase is obtained after heat treatment. However, photoluminescence measurements of SG GCs showed very promising results. Ln^3+^ incorporation in fluoride NCs was demonstrated by several authors and produced an improvement in the optical properties (linear or non-) due to the low phonon energy of the crystal hosts, thus opening the way to use SG materials for photonic applications.

On the other side, our group introduced an important modification to the SG synthesis of OxGCs. First of all, LaF_3_- and GdF_3_-based compositions with a much higher content of active phase (up to 40 mol %) have been prepared for the first time, together with NaGdF_4_ OxGCs. Moreover, new precursors and deep synthesis modification were performed by partial replacement of TEOS with MTES. 

A chemical reaction followed by fast crystal precipitation was indicated as responsible for the crystallization mechanism of fluoride NCs, as opposed to conventional diffusion-controlled processes. LaF_3_ NCs were demonstrated to be unstable with aging at crystallization or higher temperatures for a long treatment time, while for NaGdF_4_ an increase in the crystal size was observed when the treatment time increased. Therefore, the evolution and behavior of the NCs depend on the synthesis and the crystal phase. 

Rietveld refinement confirmed that a crystal fraction ~18 wt % is obtained for 80SiO_2_-20LaF_3_ GC treated at 550 °C for 1 min, such a value being the highest reported to date for SG OxGCs. 

HRTEM showed that homogeneously distributed fluoride NCs precipitate in the SiO_2_ glass matrix after fast heat treatment at 550 °C for 1 min using TEOS/MTES as SiO_2_ precursors. Moreover, EDXS confirmed dopants’ incorporation in the fluoride NCs even after fast heat treatment at 550 °C for 1 min, suggesting that dopant incorporation occurs along with NC precipitation. Such results were also confirmed by XAS measurements, revealing a fluorine-rich environment even in the xerogel sample. 

Optical measurements unambiguously showed dopant incorporation in low-phonon-energy fluoride NCs. Well-resolved Stark components and crystal-like spectra were obtained for GC samples, resulting in much higher emission intensities and more efficient ET process with respect to xerogel samples.

Considering all the results published up to now and the benefits offered by the SG method, we think that transparent SG OxGCs materials can be considered of great interest and promising for several photonic applications, but improvement of synthesis and processing is still necessary. In this sense, we hope that this paper will be useful for researchers working in this field. 

## References

[1] Feldmann C., Jüstel T., Ronda C.R., Schmidt P.J. (2003). Inorganic luminescent materials: 100 years of research and application. Adv. Funct. Mater..

[2] George N.C., Denault K.A., Seshadri R. (2013). Phosphors for solid-state white lightening. Annu. Rev. Mater. Res..

[3] Huang X. (2014). Solid-state lighting: Red phosphor converts white LEDs. Nat. Photonics.

[4] Huang X., Han S., Huang W., Liu X. (2013). Enhancing solar cell efficiency: The search for luminescent materials as spectral converters. Chem. Soc. Rev..

[5] Eliseeva S.V., Bunzli J.C.G. (2010). Lanthanide luminescence for functional materials and bio-sciences. Chem. Soc. Rev..

[6] Lee H.U., Park S.Y., Lee S.C., Choi S., Seo S., Kim H., Won J., Choi K., Kang K.S., Park H.G. (2016). Black Phosphorus (BP) nanodots for potential biomedical applications. Small.

[7] De Pablos-Martín A., Durán A., Pascual M.J. (2012). Nanocrystallisation in oxyfluoride systems: Mechanisms of crystallisation and photonic properties. Int. Mater. Rev..

[8] Fedorov P.P., Luginina A.A., Popov A.I. (2015). Transparent oxyfluoride glass ceramics. J. Fluor. Chem..

[9] De Pablos-Martín A., Ferrari M., Pascual M.J., Righini G.C. (2015). Glass-ceramics: A class of nanostructured materials for photonics. La Rivista del Nuovo Cimento.

[10] Wang Y., Ohwaki J. (1993). New transparent vitroceramics codoped with Er^3+^ and Yb^3+^ for efficient frequency upconversion. Appl. Phys. Lett..

[11] Stevenson A.J., Serier-Brault H., Gredin P., Mortier M. (2011). Fluoride materials for optical applications: Single crystals, ceramics, glasses, and glass–ceramics. J. Fluor. Chem..

[12] Lopez-Iscoa P., Salminen T., Hakkarainen T., Petit L., Janner D., Boetti N., Lastusaari M., Pugliese D., Paturi P., Milanese D. (2017). Effect of partial crystallization on the structural and luminescence properties of Er^3+^-doped phosphate glasses. Materials.

[13] Höland W., Beall G. (2002). Glass-Ceramic Technology.

[14] Augustyn E., Żelechower M., Stróż D., Chrapoński J. (2012). The microstructure of erbium-ytterbium co-doped oxyfluoride glass-ceramic optical fibers. Opt. Mater..

[15] Reben M., Dorosz D., Wasylak J., Burtan-Gwizdala B., Jaglarz J., Zontek J. (2012). Nd^3+^-doped oxyfluoride glass ceramics optical fibre with SrF_2_ nanocrystals. Opt. Appl..

[16] Krishnaiah K.V., Ledemi Y., Genevois C., Veron E., Sauvage X., Morency S., Soares de Lima Filho E., Nemova G., Allix M., Messaddeq Y. (2017). Ytterbium-doped oxyfluoride nano-glass-ceramic fibers for laser cooling. Opt. Mater. Express.

[17] Gorni G., Balda R., Fernández J., Ipparraguirre I., Velázquez J.J., Castro Y., Chen G., Sundararayan M., Pascual M.J., Durán A. (2017). Oxyfluoride glass-ceramic fibers doped with Nd^3+^: Structural and optical characterization. CrystEngComm.

[18] Roberts R.B., Tainsh R.J., White G.K. (1982). Thermal properties of Zerodur at low temperatures. Cryogenics.

[19] Owens G.J., Singh R.K., Foroutan F., Alqaysi M., Han C.M., Mahapatra C., Kim H.W., Knowles J.C. (2016). Sol-gel based materials for biomedical applications. Prog. Mater. Sci..

[20] Rywak A.A., Burlitch J.M. (1996). Sol-gel synthesis of nanocrystalline magnesium fluoride: Its use in the preparation of MgF_2_ films and MgF_2_-SiO_2_ composites. Chem. Mater..

[21] Rywak A.A., Burlitch J.M. (1996). The crystal chemistry and thermal stability of sol-gel prepared fluoride-substituted talc. Phys. Chem. Miner..

[22] Fujihara S., Tada M., Kimura T. (1997). Preparation and characterization of MgF_2_ thin film by a trifluoroacetic acid method. Thin Solid Films.

[23] Luo W., Wang Y., Bao F., Zhou L., Wang X. (2004). Crystallization behavior of PbF_2_-SiO_2_ based bulk xerogels. J. Non-Cryst. Solids.

[24] Del-Castillo J., Yanes A.C., Méndez-Ramos J., Tikhomirov V.K., Rodríguez V.D. (2009). Structure and up-conversion luminescence in sol–gel derived Er^3+^-Yb^3+^ co-doped SiO_2_:PbF_2_ nano-glass-ceramics. Opt. Mater..

[25] Del-Castillo J., Yanes A.C., Méndez-Ramos J., Tikhomirov V.K., Moshchalkov V.V., Rodríguez V.D. (2010). Sol-gel preparation and white up-conversion luminescence in rare-earth doped PbF_2_ nanocrystals dissolved in silica glass. J. Sol-Gel Sci. Technol..

[26] Szpikowska-Sroka B., Zur L., Czoik R., Goryczka T., Swinarew A.S., Żadło M., Pisarski W.A. (2013). Long-lived emission from Eu^3+^:PbF_2_ nanocrystals distributed into sol-gel silica glass. J. Sol-Gel Sci. Technol..

[27] Szpikowska-Sroka B., Pawlik N., Zur L., Czoik R., Goryczka T.M., Pisarski W.A. (2016). Effect of fluoride ions on the optical properties of Eu^3+^:PbF_2_ nanocrystals embedded into sol-gel host materials. Mater. Chem. Phys..

[28] Szpikowska-Sroka B., Pawlik N., Goryczka T., Pietrasik E., Bańczyk M., Pisarski W.A. (2017). Lead fluoride β-PbF_2_ nanocrystals containing Eu^3+^ and Tb^3+^ ions embedded in sol-gel materials: Thermal, structural and optical investigations. Ceram. Int..

[29] Yu Y., Chen D., Wang Y., Luo W., Zheng Y., Cheng Y., Zhou L. (2006). Structural evolution and its influence on luminescence of SiO_2_-SrF_2_-ErF_3_ glass ceramics prepared by sol-gel method. Mater. Chem. Phys..

[30] Yu Y., Wang Y., Chen D., Liu F. (2008). Efficient upconversion luminescence of Er^3+^:SrF_2_-SiO_2_-Al_2_O_3_ sol-gel glass ceramics. Ceram. Int..

[31] Zhou L., Chen D., Luo W., Wang Y., Yu Y., Liu F. (2007). Transparent glass ceramic containing Er^3+^:CaF_2_ nano-crystals prepared by sol-gel method. Mater. Lett..

[32] Georgescu S., Voiculescu A.M., Matei C., Secu C.E., Negre R.F., Secu M. (2013). Ultraviolet and visible up-conversion luminescence of Er^3+^/Yb^3+^ co-doped CaF_2_ nanocrystals in sol-gel derived glass-ceramics. J. Lumin..

[33] Jiang Y., Fan J., Jiang B., Mao X., Zhou C., Zhang L. (2016). Structure and optical properties of transparent Er^3+^-doped CaF_2_-silica glass ceramic prepared by controllable sol-gel method. Ceram. Int..

[34] Chen D., Wang Y., Yu Y., Ma E., Zhou L. (2006). Microstructure and luminescence of transparent glass ceramic containing Er^3+^:BaF_2_ nano-crystals. J. Solid State Chem..

[35] Secu C.E., Secu M., Ghica C., Mihut L. (2011). Rare-earth doped sol-gel derived oxyfluoride glass-ceramics: Structural and optical characterization. Opt. Mater..

[36] Secu C.E., Bartha C., Polosan S., Secu M. (2014). Thermally activated conversion of a silicate gel to an oxyfluoride glass ceramic: Optical study using Eu^3+^ probe ion. J. Lumin..

[37] Fujihara S., Kitta S., Kimura T. (2003). Porous Phosphor thin films of oxyfluoride SiO_2_-BaMgF_4_: Eu^2+^ glass-ceramics prepared by sol-gel method. Chem. Lett..

[38] Kitta S., Fujihara S., Kimura T. (2004). Porous SiO_2_-BaMgF_4_:Eu(II) glass-ceramic thin films and their strong blue photoluminescence. J. Sol-Gel Sci. Technol..

[39] Blasse G., van den Heuvel G.P.M., Van Dijk T. (1979). Energy transfer from Gd^3+^ to Tb^3+^ and Eu^3+^. Chem. Phys. Lett..

[40] Grzyb T., Runowski M., Lis S. (2014). Facile synthesis, structural and spectroscopic properties of GdF_3_:Ce^3+^, Ln^3+^ (Ln^3+^ = Sm^3+^, Eu^3+^, Tb^3+^, Dy^3+^) nanocrystals with bright multicolor luminescence. J. Lumin..

[41] Pokhrel M., Mimun L.C., Yust B., Kumar G.A., Dhanale A., Tang L., Sardara D.K. (2014). Stokes emission in GdF_3_:Nd^3+^ nanoparticles for bioimaging probes. Nanoscale.

[42] Fujihara S., Tada M., Kimura T. (1998). Sol-gel processing of LaF_3_ thin films. J. Ceram. Soc. JPN.

[43] Fujihara S., Tada M., Kimura T. (1999). Formation of LaF_3_ microcrystals in sol-gel silica. J. Non-Cryst. Solids.

[44] Tada M., Fujihara S., Kimura T. (1999). Sol-gel processing and characterization of alkaline earth and rare-earth fluoride thin films. J. Mater. Res..

[45] Fujihara S., Kato T., Kimura T. (2000). Influence of solution composition on the formation of SiO_2_/LaF_3_ composites in the sol-gel process. J. Mater. Sci..

[46] Fujihara S., Tada M., Kimura T. (2000). Controlling factors for the conversion of trifluoroacetate sols into thin metal fluoride coatings. J. Sol-Gel Sci. Technol..

[47] Fujihara S., Kato T., Kimura T. (2001). Sol-gel synthesis of silica-based oxyfluoride glass-ceramic thin films: incorporation of Eu^3+^ activators into crystallites. J. Am. Ceram. Soc..

[48] Ribeiro S.J.L., Araújo C.C., Bueno L.A., Gonçalves R.R., Messaddeq Y. (2004). Sol-gel Eu^3+^/Tm^3+^ doped transparent glass-ceramic waveguides. J. Non-Cryst. Solids.

[49] Biswas A., Maciel G.S., Friend C.S., Prasad P.N. (2003). Upconversion properties of a transparent Er^3+^-Yb^3+^co-doped LaF_3_-SiO_2_ glass-ceramics prepared by sol-gel method. J. Non-Cryst. Solids.

[50] Yanes A.C., del-Castillo J., Méndez-Ramos J., Rodríguez V.D., Torres M.E., Arbiol J. (2007). Luminescence and structural characterization of transparent nanostructured Eu^3+^-doped LaF_3_-SiO_2_ glass-ceramics prepared by sol-gel method. Opt. Mater..

[51] Velázquez J.J., Yanes A.C., del Castillo J., Méndez-Ramos J., Rodríguez V.D. (2007). Optical properties of Ho^3+^-Yb^3+^ co-doped nanostructured SiO_2_-LaF_3_ glass-ceramics prepared by sol-gel method. Phys. Status Solidi A.

[52] Méndez-Ramos J., Velázquez J.J., Yanes A.C., del Castillo J., Rodríguez V.D. (2008). Up-conversion in nanostructured Yb^3+^-Tm^3+^ co-doped sol-gel derived SiO_2_-LaF_3_ transparent glass-ceramics. Phys. Status Solidi A.

[53] Yanes A.C., Velázquez J.J., del Castillo J., Méndez-Ramos J., Rodríguez V.D. (2009). Colour tuneability and white light generation in Yb^3+^-Ho^3+^-Tm^3+^ co-doped SiO_2_-LaF_3_ nano-glass-ceramics prepared by sol-gel method. J. Sol-Gel Sci. Technol..

[54] Velázquez J.J., Rodríguez V.D., Yanes A.C., del Castillo J., Méndez-Ramos J. (2010). Increase in the Tb^3+^ green emission in SiO_2_-LaF_3_ nano-glass-ceramics by codoping with Dy^3+^ ions. J. Appl. Phys..

[55] Velázquez J.J., Rodríguez V.D., Yanes A.C., del Castillo J., Méndez-Ramos J. (2012). Photon down-shifting by energy transfer from Sm^3+^ to Eu^3+^ ions in sol-gel SiO_2_-LaF_3_ nano-glass-ceramics for photovoltaics. Appl. Phys. B.

[56] Dejneka M.J. (1998). The luminescence and structure of novel transparent oxyfuoride glass-ceramics. J. Non-Cryst. Solids.

[57] Luo W., Wang Y., Cheng Y., Bao F., Zhou L. (2006). Crystallization and structural evolution of YF_3_-SiO_2_ xerogel. Mater. Sci. Eng. B.

[58] Méndez-Ramos J., Santana-Alonso A., Yanes A.C., del Castillo J., Rodríguez V.D. (2010). Rare-earth doped YF_3_ nanocrystals embedded in sol-gel silica glass matrix for white light generation. J. Lumin..

[59] Santana-Alonso A., Méndez-Ramos J., Yanes A.C., del Castillo J., Rodríguez V.D. (2010). White light up-conversion in transparent sol-gel derived glass-ceramics containing Yb^3+^-Er^3+^-Tm^3+^ triply-doped YF_3_ nanocrystals. Mater. Chem. Phys..

[60] Yanes A.C., Santana-Alonso A., Méndez-Ramos J., del Castillo J., Rodríguez V.D. (2011). Novel sol-gel nano-glass-ceramics comprising Ln^3+^-Doped YF_3_ nanocrystals: structure and high efficient UV up-conversion. Adv. Funct. Mater..

[61] Chen D., Wang Y., Yu Y., Huang P. (2008). Structure and optical spectroscopy of Eu-doped glass ceramics containing GdF_3_ nanocrystals. J. Phys. Chem. C.

[62] Shan Z., Chen D., Yu Y., Huang P., Lin H., Wang Y. (2010). Luminescence in rare earth-doped transparent glass ceramics containing GdF_3_ nanocrystals for lighting applications. J. Mater. Sci..

[63] Yin W., Zhao L., Zhou L., Gu Z., Liu X., Tian G., Jin S., Yan L., Ren W., Xing G., Zhao Y. (2012). Enhanced red emission from GdF_3_:Yb^3+^, Er^3+^ upconversion nanocrystals by Li^+^ doping and their application for bioimaging. Chem. Eur. J..

[64] Fujihara S., Koji S., Kimura T. (2004). Structure and optical properties of (Gd,Eu)F_3_-nanocrystallized sol-gel silica films. J. Mater. Chem..

[65] Szpikowska-Sroka B., Zur L., Czoik R., Goryczka T., Żądło M., Pisarski W.A. (2014). Ultraviolet-to-visible downconversion luminescence in solgel oxyfluoride glass ceramics containing Eu^3+^:GdF_3_ nanocrystals. Opt. Lett..

[66] Szpikowska-Sroka B., Pawlik N., Goryczka T., Pisarski W.A. (2015). Influence of silicate sol-gel host matrices and catalyst agents on the luminescent properties of Eu^3+^/Gd^3+^ under different excitation wavelengths. RSC Adv..

[67] Pawlik N., Szpikowska-Sroka B., Sołtys M., Pisarski W.A. (2016). Optical properties of silica sol-gel materials singly- and doubly-doped with Eu^3+^and Gd^3+^ ions. J. Rare Earth.

[68] Kano T., Yamamoto H., Otomo Y. (1972). NaLnF_4_: Yb^3+^, Er^3+^ (Ln: Y, Gd, La): Efficient green-emitting infrared-excited phosphors. J. Electrochem. Soc..

[69] Krämer K.W., Biner D., Frei G., Güdel H.U., Hehlen M.P., Lüthi S.R. (2004). Hexagonal sodium yttrium fluoride based green and blue emitting upconversion phosphors. Chem. Mater..

[70] Liu F., Ma E., Chen D., Yu Y., Wang Y. (2006). Tunable red-green upconversion luminescence in novel transparent glass ceramics containing Er: NaYF_4_ nanocrystals. J. Phys. Chem. B.

[71] De Pablos-Martín A., Méndez-Ramos J., del Castillo J., Durán A., Rodríguez V.D., Pascual M.J. (2015). Crystallization and up-conversion luminescence properties of Er^3+^/Yb^3+^-doped NaYF_4_-based nano-glass-ceramics. J. Eur. Ceram. Soc..

[72] Yanes A.C., Santana-Alonso A., Méndez-Ramos J., del Castillo J., Rodríguez V.D. (2009). Yb^3+^-Er^3+^ co-doped sol-gel transparent nano-glass-ceramics containing NaYF_4_ nanocrystals for tuneable up-conversion phosphors. J. Alloy. Compd..

[73] Santana-Alonso A., Yanes A.C., Méndez-Ramos J., del Castillo J., Rodríguez V.D. (2010). Sol-gel transparent nano-glass-ceramics containing Eu^3+^-doped NaYF_4_ nanocrystals. J. Non-Cryst. Solids.

[74] Santana-Alonso A., Méndez-Ramos J., Yanes A.C., del Castillo J., Rodríguez V.D. (2010). Up-conversion in sol-gel derived nano-glass-ceramics comprising NaYF_4_ nano-crystals doped with Yb^3+^, Ho^3+^ and Tm^3+^. Opt. Mater..

[75] Méndez-Ramos J., Yanes A.C., Santana-Alonso A., del Castillo J., Rodríguez V.D. (2010). Colour tuneability in sol-gel nano-glass-ceramics comprising Yb^3+^-Er^3+^-Tm^3+^ Co-Doped NaYF_4_ nanocrystals. J. Nanosci. Nanotechno..

[76] Méndez-Ramos J., Yanes A.C., Santana-Alonso A., del Castillo J. (2013). Highly efficient up-conversion and bright white light in RE co-doped KYF_4_ nanocrystals in sol-gel silica matrix. Chem. Phys. Lett..

[77] Yanes A.C., Santana-Alonso A., Méndez-Ramos J., del Castillo J. (2013). Structure and intense UV up-conversion emissions in RE^3+^-doped sol-gel glass-ceramics containing KYF_4_ nanocrystals. Appl. Phys. B.

[78] Del Castillo J., Yanes A.C., Santana-Alonso A., Méndez-Ramos J. (2014). Efficient dual-wavelength excitation of Tb^3+^ emission in rare-earth doped KYF_4_ cubic nanocrystals dispersed in silica sol-gel matrix. Opt. Mater..

[79] Yanes A.C., del Castillo J. (2016). Enhanced emission via energy transfer in RE co-doped SiO_2_-KYF_4_ nano-glass-ceramics for white LEDs. J. Alloy. Compd..

[80] Deng D., Xu S., Zhao S., Li C., Wang H., Ju H. (2009). Enhancement of upconversion luminescence in Tm^3+^/Er^3+^/Yb^3+^-codoped glass ceramic containing LiYF_4_ nanocrystals. J. Lumin..

[81] Kawamura G., Yoshimura R., Ota K., Oh S.Y., Hakiri N., Muto H., Hayakawa T., Matsuda A. (2013). A unique approach to characterization of sol-gel-derived rare-earth-doped oxyfluoride glass-ceramics. J. Am. Ceram. Soc..

[82] Secu C.E., Negrea R.F., Secu M. (2013). Eu^3+^ probe ion for rare-earth dopant site structure in sol-gel derived LiYF_4_ oxyfluoride glass-ceramic. Opt. Mater..

[83] Kawamura G., Yoshimura R., Ota K., Oh S.Y., Muto H., Hayakawa T., Matsuda A. (2013). Extraction of Nd^3+^-doped LiYF_4_ phosphor from sol-gel-derived oxyfluoride glass ceramics by hydrofluoric acid treatment. Opt. Mater..

[84] Secu M., Secu C.E. (2015). Up-conversion luminescence of Er^3+^/Yb^3+^ co-doped LiYF_4_ nanocrystals in sol-gel derived oxyfluoride glass-ceramics. J. Non-Cryst. Solids.

[85] Del Castillo J., Yanes A.C., Abe S., Smet P.F. (2015). Site selective spectroscopy in BaYF_5_:RE^3+^ (RE = Eu, Sm) nano-glass-ceramics. J. Alloy. Compd..

[86] Del Castillo J., Yanes A.C. (2017). Bright luminescence of Gd^3+^ sensitized RE^3+^-doped SiO_2_-BaGdF_5_ glass-ceramics for UV-LEDs colour conversion. J. Alloy. Compd..

[87] Chiodini N., Paleari A., DiMartino D., Spinolo G. (2022). SnO_2_ nanocrystals in SiO_2_ : A wide-band-gap quantum-dot system. Appl. Phys. Lett..

[88] Du J., Zhao R., Xie Y., Li J. (2015). Size-controlled synthesis of SnO_2_ quantum dots and their gas-sensing performance. Appl Surf. Sci..

[89] Nogami M., Enomoto T., Hayakawa T. (2002). Enhanced fluorescence of Eu^3+^ induced by energy transfer from nanosized SnO_2_ crystals in glass. J. Lumin..

[90] Chiodini N., Paleari A., Spinolo G., Crespi P. (2003). Photorefractivity in SiO_2_:SnO_2_ glass-ceramics by visible light. J. Non-Cryst. Solids.

[91] Yanes A.C., del Castillo J., Torres M., Peraza J., Rodríguez V.D., Méndez-Ramos J. (2004). Nanocrystal-size selective spectroscopy in SnO^2^: Eu^3+^ semiconductor quantum dots. Appl. Phys. Lett..

[92] Del Castillo J., Rodríguez V.D., Yanes A.C., Méndez-Ramos J., Torres M.E. (2005). Luminescent properties of transparent nanostructured Eu^3+^ doped SnO_2_–SiO_2_ glass-ceramics prepared by the sol-gel method. Nanotechnology.

[93] Del Castillo J., Yanes A.C., Velázquez J.J., Méndez-Ramos J., Rodríguez V.D. (2009). Luminescent properties of Eu^3+^-Tb^3+^-doped SiO_2_-SnO_2_-based nano-glass-ceramics prepared by sol-gel method. J. Alloy. Compd..

[94] Morais E.A., Ribeiro S.J.L., Scalvi L.V.A., Santilli C.V., Ruggiero L.O., Pulcinelli S.H., Messadeq Y. (2002). Optical characteristics of Er^3+^-Yb^3+^ doped SnO_2_ xerogels. J. Alloy. Compd..

[95] Van Tran T.T., Si Bui T., Turrell S., Capoen B., Roussel P., Bouazaoui M., Ferrari M., Cristini O., Kinowski C. (2012). Controlled SnO_2_ nanocrystal growth in SiO_2_-SnO_2_ glass-ceramic monoliths. J. Raman Spectrosc..

[96] Van Tran T.T., Turrell S., Capoen B., Le Van H., Ferrari M., Ristic D., Boussekey L., Kinowski C. (2014). Environment segregation of Er^3+^ emission in bulk sol-gel-derived SiO_2_-SnO_2_ glass ceramics. J. Mater. Sci..

[97] Gonçalves R.R., Messaddeq Y., Chiasera A., Jestin Y., Ferrari M., Ribeiro S.J.L. (2008). Erbium-activated silica-zirconia planar waveguides prepared by sol-gel route. Thin Solid Films.

[98] Gonçalves R.R., Guimarães J.J., Ferrari J.L., Maia L.J.Q., Ribeiro S.J.L. (2008). Active planar waveguides based on sol-gel Er^3+^-doped SiO_2_-ZrO_2_ for photonic applications: Morphological, structural and optical properties. J. Non-Cryst. Solids.

[99] Suhaimi N.F.M., Rashid S.N.M., Junit N.F.H.M., Iznie Razakia N., Abd-Rahmana M.K., Ferrari M. Effect of Zirconia in Er^3+^-doped SiO_2_-ZrO_2_ for planar waveguide laser. Proceedings of the 2012 IEEE 3rd International Conference on Photonics (ICP).

[100] Dos Santos Cunha C., Ferrari J.L., de Oliveira D.C., Maia L.J.Q., Gomes A.S.L., Ribeiro S.J.L., Gonçalves R.R. (2012). NIR luminescent Er^3+^/Yb^3+^ co-doped SiO_2_-ZrO_2_ nanostructured planar and channel waveguides: Optical and structural properties. Mater. Chem. Phys..

[101] Ribeiro S.J.L., Messaddeq Y., Gonçalves R.R., Ferrari M., Montagna M., Aegerter M.A. (2000). Low optical loss planar waveguides prepared in an organic-inorganic hybrid system. Appl. Phys. Lett..

[102] Jestin Y., Armellini C., Chiappini A., Chiaser A., Ferrari M., Goyes C., Montagna M., Moser E., Nunzi Conti G., Pelli S. (2007). Erbium activated HfO_2_ based glass-ceramics waveguides for photonics. J. Non-Cryst. Solids.

[103] Ferrari J.L., Lima K.O., Maia L.J.Q., Ribeiro S.J.L., Gonçalves R.R. (2011). Structural and spectroscopic properties of luminescent Er^3+^-Doped SiO_2_-Ta_2_O_5_ nanocomposites. J. Am. Ceram. Soc..

[104] Aquino F.T., Ferrari J.L., Ribeiro S.J.L., Ferrier A., Goldner P., Gonçalves R.R. (2013). Broadband NIR emission in novel sol-gel Er^3+^-doped SiO_2_-Nb_2_O_5_ glass ceramic planar waveguides for photonic applications. Opt. Mater..

[105] Secu M., Secu C.E., Bartha C. (2016). Crystallization and luminescence properties of a new Eu^3+^-doped LaOCl nano-glass-ceramic. J. Eur. Ceram. Soc..

[106] Innocenzi P., Abdirashid M.O., Guglielmi M. (1994). Structure and properties of sol-gel coatings from methyltriethoxysilane and tetraethoxysilane. J. Sol-Gel Sci. Technol..

[107] Ravel B., Newville M. (2005). ATHENA, ARTEMIS, HEPHAESTUS: Data analysis for X-ray absorption spectroscopy using IFEFFIT. J. Synchrotron Radiat..

[108] Ma Y., Lee H.R., Tsuru T. (2012). Study on preparation and hydrophobicity of MTES derived silica sol and gel. Adv. Mater. Res..

[109] Jia Y.Q. (1991). Crystal Radii and effective ionic radii of the rare earth ions. J. Sol. State Chem..

[110] Gorni G., Pascual M.J., Caballero A., Velázquez J.J., Mosa J., Castro Y., Durán A. (2018). Crystallization mechanism in sol-gel oxyfluoride glass-ceramics. J. Non-Cryst. Solids.

[111] Gorni G., Balda R., Ferández J., Velázquez J.J., Pascual L., Mosa J., Durán A., Castro Y. (2017). 80SiO_2_-20LaF_3_ oxyfluoride glass ceramic coatings doped with Nd^3+^ for optical applications. Int. J. Appl. Glass Sci..

[112] Velázquez J.J., Mosa J., Gorni G., Balda R., Ferández J., Pascual L., Durán A., Castro Y. (2018). Transparent SiO_2_-GdF_3_ sol-gel nano-glass ceramics for optical applications. J. Sol-Gel Sci. Technol..

[113] Chen D., Yu Y., Huang P., Wang Y. (2009). Nanocrystallization of lanthanide trifluoride in an aluminosilicate glass matrix: Dimorphism and rare earth partition. CrystEngComm.

[114] Bartha C., Secu C.E., Matei E., Secu M. (2017). Crystallization kinetics mechanism investigation of sol-gel-derived NaYF_4_:(Yb,Er) up-converting phosphors. CrystEngComm.

[115] Agarwal B.K., Verma L.P. (1970). A rule for chemical shifts of X-ray absorption edges. J. Phys. C Solid State.

[116] Gorni G., Velázquez J.J., Mather G.C., Durán A., Chen G., Sundararaja M., Balda R., Ferández J., Pascual M.J. (2017). Selective excitation in transparent oxyfluoride glass-ceramics doped with Nd^3+^. J. Eur. Ceram. Soc..

